# Pool-GWAS on reproductive dormancy in *Drosophila simulans* suggests a polygenic architecture

**DOI:** 10.1093/g3journal/jkac027

**Published:** 2022-02-07

**Authors:** Manolis Lirakis, Viola Nolte, Christian Schlötterer

**Affiliations:** 1 Institut für Populationsgenetik, Vetmeduni Vienna, 1210 Wien, Austria; 2 Vienna Graduate School of Population Genetics, Vetmeduni Vienna, 1210 Wien, Austria

**Keywords:** dormancy, *Drosophila*, adaptation, genetic architecture

## Abstract

The genetic basis of adaptation to different environments has been of long-standing interest to evolutionary biologists. Dormancy is a well-studied adaptation to facilitate overwintering. In *Drosophila melanogaster*, a moderate number of genes with large effects have been described, which suggests a simple genetic basis of dormancy. On the other hand, genome-wide scans for dormancy suggest a polygenic architecture in insects. In *D. melanogaster*, the analysis of the genetic architecture of dormancy is complicated by the presence of cosmopolitan inversions. Here, we performed a genome-wide scan to characterize the genetic basis of this ecologically extremely important trait in the sibling species of *D. melanogaster*, *D. simulans* that lacks cosmopolitan inversions. We performed Pool-GWAS in a South African *D. simulans* population for dormancy incidence at 2 temperature regimes (10 and 12°C, LD 10:14). We identified several genes with SNPs that showed a significant association with dormancy (*P*-value < 1e-13), but the overall modest response suggests that dormancy is a polygenic trait with many loci of small effect. Our results shed light on controversies on reproductive dormancy in *Drosophila* and have important implications for the characterization of the genetic basis of this trait.

## Introduction

Organisms are regularly exposed to unfavorable stressful conditions, but genetic adaptations can reduce their impact and increase fitness. The *Drosophila melanogaster* species subgroup provides great models to study adaptation to stressful environmental conditions. Members of the *D.melanogaster* subgroup originated in sub-Saharan Africa and nearby islands (e.g. *D. simulans* from Madagascar), and subsequently colonized temperate habitats in Eurasia and more recently, North America and Australia (David and Capy 1988; Dean and Ballard 2004; Cogni et al. 2014). Latitudinal and seasonal clines spanning temperate to subtropical/tropical regions for phenotypes and genomic variation reflect adaption to spatially varying selection (e.g. David *et al.* 1985; Berry and Kreitman 1993; Arthur *et al.* 2008; Fabian *et al.* 2012; Bergland *et al.* 2014; Behrman *et al.* 2015; Machado *et al.* 2016). Given the abundant molecular, genetic, and genomic resources available not only for *D. melanogaster*, but also sister species, e.g. *D. simulans*, these species provide an excellent opportunity to study adaptation to novel heterogeneous environments.

Winter is a particularly stressful condition for insects, when temperature drops dramatically and feeding resources become scarce. Dormancy is an important adaptation to facilitate overwintering. It is a state of suppressed development, reproduction, metabolic activities, and senescence (Denlinger 2002; Hahn and Denlinger 2011), which allows the organism to “escape in time” until the environmental conditions are favorable again (Williams and Sokolowski 1993; Tatar and Yin 2001; Zonato *et al.* 2017). The ability of *D. melanogaster* to overwinter is well studied. They overwinter as adults (Izquierdo 1991; Mitrovski and Hoffmann 2001; Hoffmann *et al.* 2003; Strachan *et al.* 2011; Stephens *et al.* 2015) by expressing a reproductive dormancy at low temperatures and/or short photoperiods (e.g. Saunders *et al.* 1989; Williams and Sokolowski 1993; Tatar *et al.* 2001; Schmidt *et al.* 2005; Schmidt and Conde 2006; Baker and Russell 2009; Emerson *et al.* 2009b; Lee *et al.* 2011; Zonato *et al.* 2017; Anduaga *et al.* 2018; Lirakis *et al.* 2018); dormant adult female flies have underdeveloped ovaries through the mid-oogenesis checkpoint, reduced metabolism, delayed senescence, and elevated stress resistance (Tauber *et al.* 1986; Tatar *et al.* 2001; Schmidt and Conde 2006; Kubrak *et al.* 2014; Lirakis *et al.* 2018).

It has been known for a long time that dormancy in *D*. *melanogaster* and other insects is regulated by juvenile hormone, ecdysteroid and insulin signaling (Saunders *et al.* 1989, 1990; Gilbert *et al.* 1998; Richard *et al.* 2001, 2005; Tatar *et al.* 2001; Denlinger 2002; Emerson *et al.* 2009a; Denlinger *et al.* 2012; Sim and Denlinger 2013; Kubrak *et al.* 2014; Santos *et al.* 2019; Guo *et al.* 2021; Hasebe and Shiga 2021). In *D*. *melanogaster*, variation of the syndrome has been linked to a small number of genes including the insulin-regulated *PI3-kinase* (*Dp110*) (Williams *et al.* 2006), *timeless* (Sandrelli *et al.* 2007), and *couch potato* (*cpo*) (Schmidt *et al.* 2008; Cogni *et al.* 2014). The role of insulin signaling has been further demonstrated by blocking the production of *Drosophila* insulin-like peptides (Liu *et al.* 2016; Schiesari *et al.* 2016) or through insulin-producing cells inactivation (Ojima *et al.* 2018). With only few genes being reported, which have a substantial effect on dormancy, these studies suggest that the syndrome in *D*. *melanogaster* is either a simple trait or a few major loci are acting synergistically with a polygenic background.

On the other hand, dormancy is a complex trait that involves many physiological processes, which can be grouped into 3 main categories: (1) the perception of environmental stimuli, (2) hormonal signaling, and (3) and expression of the syndrome by blocking oogenesis (Allen 2007; Emerson *et al.* 2009a). In concordance with the complex nature of the trait, several genome-wide scans in insects suggested a polygenic architecture for dormancy—i.e. many loci each with very small effect sizes (Ragland *et al.* 2017; Pruisscher *et al.* 2018; Kauranen *et al.* 2019; Ragland *et al.* 2019). In *D. melanogaster*, the interpretation of results from genome-wide association studies (GWAS) is complicated due to the presence of cosmopolitan inversions that suppress recombination and complicate the mapping of causative variants due to linkage (Aulard *et al.* 2004). *cpo*, a gene for which a significant effect on dormancy distribution was demonstrated in populations from the US East Coast (Schmidt *et al.* 2008; Cogni *et al.* 2014), lies within the inversion *In(3R)Payne* that is distributed clinally in that area (Knibb 1982; Sezgin *et al.* 2004; Fabian *et al.* 2012; Kapun *et al.* 2014). However, the role of *cpo* as a key gene in dormancy evolution has been questioned in European populations where *In(3R)Payne* does not appear to be clinally distributed (Zonato *et al.* 2016) and in Australian populations after the inversion’s clinal distribution was taken into account (Lee *et al.* 2011). Interestingly, a genome-wide association study for dormancy in *D. melanogaster* in an American *D. melanogaster* population did not confirm *cpo* as a candidate gene for dormancy, but suggested a polygenic architecture for the trait (Erickson *et al.* 2020).

A recent multipopulation analysis of seasonal variation in *D. melanogaster* showed that only genomic regions associated with inversions were enriched for seasonally fluctuating SNPs (Machado *et al.* 2021). As dormancy-causative variants are assumed to fluctuate seasonally in *D. melanogaster* (Schmidt and Conde 2006; Bergland *et al.* 2014), it is possible that dormancy-related alleles are located in the inversions, and the suppressed recombination creates “super alleles” favored at dormancy and nondormancy conditions. It is, however, not clear how strong the association between inversion frequency and dormancy incidence is, as only modest association between inversion frequency and dormancy incidence was observed (Erickson *et al.* 2020). It is possible that alleles associated with the inversion may appear as major effect loci, in particular when the inversion status is not taken into account. On the other hand, when the inversion status is included in the analysis, only polygenic signatures may be detected because it is difficult to disentangle the presence/absence of inversions from the effect of contributing loci with large effect.

Given these complications caused by segregating inversions, we scrutinized the genetic architecture of reproductive dormancy in *D. simulans*, a close relative of *D. melanogaster*. Similar to *D. melanogaster*, *D. simulans* enters a dormant state under low temperatures and short photoperiods (Zonato *et al.* 2017; Lirakis *et al.* 2018). However, unlike *D. melanogaster*, where inversions are common, *D. simulans* has no cosmopolitan inversions segregating in natural populations (Aulard *et al.* 2004). This does not only facilitate GWAS and studies of adaptation based on genomic signatures (Barghi *et al.* 2017), but also allows us to determine the genetic basis of dormancy without the confounding effect of seasonally fluctuating inversions.

## Materials and methods

### Dormancy phenotyping

For dormancy screening and genetic analysis, we used a single *D. simulans* population, as the homogeneous genetic background is crucial—genetic stratification among the studied population could compromise our genetic analysis. Flies were collected from a natural *D. simulans* population collected near Stellenbosch, South Africa in 2013 March. In this area, temperatures drop well below 10°C during winter, so flies are expected to express a reproductive dormancy to deal with these conditions. Furthermore, Zonato *et al.* (2017) and Lirakis *et al.* (2018) found that African fly populations express dormancy. More than 1,000 isofemale strains were established by placing single, freshly collected females in a food vial. These isofemale strains were maintained under standard laboratory conditions for more than 4 years prior to the dormancy assays. We screened this South-African population following the protocol of Lirakis *et al.* (2018) at 10 and 12°C dormancy-inducing conditions (LD 10:14). These 2 temperatures represent the range where dormancy variation is observed within and between strains (Zonato *et al.* 2017; Lirakis *et al.* 2018). Dormancy incidence increases with decreasing temperature and the difference in dormancy incidence between the 2 temperatures reflects plasticity that varies between strains. Given this plasticity, we used the phenotypic information from both temperatures reasoning that this can allow for a more detailed genetic dissection of the trait.

Following standard protocols in *Drosophila* to eliminate transgenerational effects (Schmidt and Conde 2006; Charette *et al.* 2011; Yampolsky *et al.* 2012; Hollis *et al.* 2014; Kellermann *et al.* 2015; Graves *et al.* 2017; Maclean *et al.* 2018; Mallard *et al.* 2018; Barghi *et al.* 2019; Hsu *et al.* 2019; Sutter *et al.* 2019; Jakšić *et al.* 2020), we screened dormancy in flies with controlled age and density of their parental generation. Both phenotyped and parental generations were maintained at 23°C, LD 12:12. For dormancy screening, freshly eclosed flies were transferred to dormancy-inducing conditions for 3 weeks before dissection. After dissecting the abdomen, the head and thorax remnants of the dissected flies were stored in ethanol in a −80°C freezer for subsequent DNA extraction. We inferred the number of flies to be phenotyped for a reliable phenotypic inference by re-analyzing phenotypic data of a number of individual strains from the South-African *D. simulans* population (Lirakis *et al.* 2018). Using the equation:

n= log (1−p)/ log (1−a),

where *n* is the number of flies, *p* is the minimum statistical power, and *a* is the frequency of the minor phenotype, we concluded that at least 13 flies per isofemale strain per temperature should be examined to infer the dormancy phenotypes with > 80% accuracy.

### Dormancy classification

Following the dormancy classification suggested by Lirakis *et al.* (2018), we defined dormancy level per strain as the fraction of flies that blocked oogenesis up to early vitellogenic egg chambers (i.e. up to stage 9 of oogenesis). We also calculated the average number of eggs produced by each strain using only the flies that produced eggs. We compared the dormancy levels and average number of eggs between the 2 temperature regimes with 2 independent Wilcoxon signed-rank tests. While estimating heritability for each temperature regime using these phenotypic data would be interesting, we caution that the extent of inbreeding in the isofemale strains was not known. Hence, we refrained from estimating heritability, but compared the within and between-strain variation for each temperature regime (R package variancePartition, function fitExtractVarPartModel) (Hoffman and Schadt 2016; Hoffman and Roussos 2021).

**Fig. 1.**
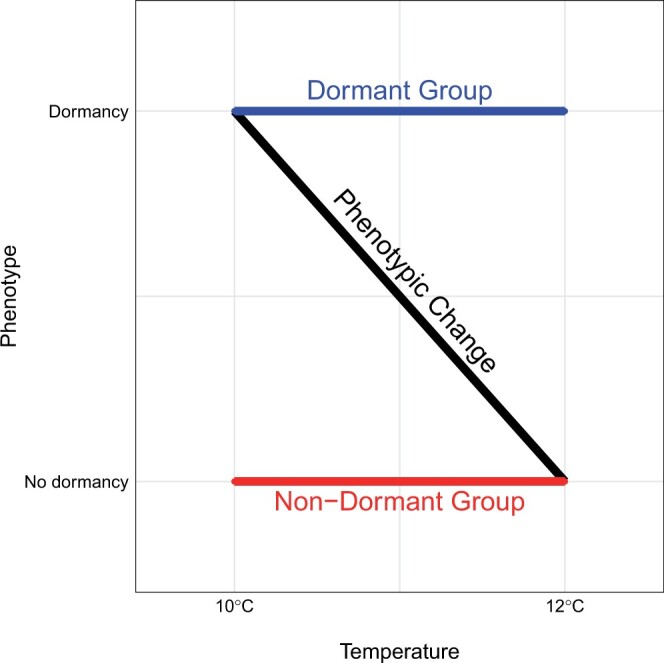
Experimental design for the dormancy Pool-GWAS. Isofemale strains that are nondormant at 10°C (thus nondormant at both temperatures since dormancy incidence decreases from 10 to 12°C) are referred to as the “Non-Dormant Group.” Isofemale strains that are dormant at 12°C (thus dormant at both temperatures since dormancy incidence increases from 12 to 10°C) constitute the “Dormant Group.” The black diagonal line represents the expected change in dormancy incidence between the 2 temperature regimes.

We performed a GWAS using sequencing of pools of individuals (Pool-Seq) with extreme phenotypes (Bastide *et al.* 2013; Schlötterer *et al.* 2014). We identified strains with extreme dormancy phenotypes according to Fig. 1:

- “Non-Dormant Group” (ND): strains at the far nondormant end (dormancy levels close to 0%) of the distribution at 10°C (thus nondormant at both temperatures since dormancy incidence decreases from 10 to 12°C).
- “Dormant Group” (D): strains at the far dormant end (dormancy levels close to 100%) of the distribution at 12°C (thus dormant at both temperatures since dormancy incidence increases from 12 to 10°C).

These groups were identified by Principal Component Analysis of the dormancy levels at 10 and 12°C (PCA of 2 phenotypes) [R package stats, function prcomp (R Core Team 2021) and visualized with the R package factoextra, function fviz_pca_ind (Kassambara and Mundt 2020)].

Subsequently, we created 4 replicate pools for each group with an extreme phenotype. For each phenotypic group, we used 1 dissected fly (with the respective phenotype) from the corresponding isofemale strains to generate a replicate pool. Females from the most extreme 25 isofemale strains were used to generate each replicate pool. Library preparation and pool sequencing are described in Supplementary File 2.

### Association analysis

We reasoned that the 4 replicates of each extreme group were not truly independent, as the replicate flies of each strain are actually related to each other. Hence, after processing the sequencing data (Supplementary File 2), we merged the replicates of each extreme group. We down-sampled the processed, filtered mapped data (bam files) of each library to 30,000,000 reads (slightly below the smallest library with 31,741,963 reads) with samtools (command view, option –s) and merged them accordingly with picard (tool MergeSamFiles). The final bam files were converted to an mpileup file using samtools and then to a synchronized pileup file using PoPoolation2, and regions with repeats and TEs were removed from this file as described in the Supplementary File 2.

The data were loaded in R with the R package poolSeq (functions read.sync, coverage, and af) (Taus *et al.* 2017), that extracts biallelic counts. Pairwise genome-wide associations were inferred with a standard chi-squared test. For SNP calling, we considered only positions with a minimum coverage of 15 reads per group and a minor allele count greater than 7 in at least one of the 2 groups. We further removed the 2% most highly covered positions. For the SNPs that passed these filtering criteria, we calculated the natural logarithm of the odds ratio as a proxy for the SNP effects, using the Haldane–Anscombe correction to account for zero counts (Anscombe 1956; Haldane 1956).

For multiple testing correction, we applied a false discovery rate (FDR) method that takes over-dispersion into account, by using the distribution of *P*-values from the adjusted chi-squared test under the null hypothesis, according to Bastide *et al.* (2013) (Supplementary File 2). In addition, we applied a permutation-based approach to determine how likely it is to obtain *P*-values as low or lower than in the chi-squared test of the original data by chance. We shuffled the D/ND labels of the 8 libraries prior merging the replicates of each extreme group, creating 34 additional datasets. These datasets were analyzed as described in the previous paragraph.

### Structural polymorphisms analysis

We further specifically searched for structural polymorphisms associated with the trait. We reasoned that regions appearing in multiple copies in our data may collapse on each other on the reference assembly if only 1 copy is present in the assembly. This would lead to differences in coverage and allele frequencies. To search for such differences, we repeated the sequencing data processing and association analysis pipeline described above, but omitted the RepeatMasker step and included positions with high coverage. We identified differences in coverage by searching for genome-wide differences in the mean coverage of 200 bp windows between the 2 extreme dormancy groups (R package poolSeq, functions read.sync and coverage). Differences in allele frequencies were detected by the adjusted chi-squared test described above. However, since we used Pool-Seq data, copy number variation in only few individuals from a pool can already result in a moderate coverage increase. To investigate whether the observed copy number differences between the 2 groups were the result of such an artifact, we sequenced single phenotyped female flies from each of 12 strains from the nondormant group. We chose flies with the nondormant phenotype for individual sequencing since we detected 2 significant regions of high coverage that were specific to the nondormant group. Library preparation, sequencing of individual flies, and sequencing data processing are described in Supplementary File 2.

### Gene ontology enrichment analysis

Gene ontology (GO) enrichment of the top SNPs was performed by Gowinda (Kofler and Schlötterer 2012), based on the M252 annotation (Palmieri *et al.* 2015), the GO from Bioconductor (package GO.db, object GOTERM) (Carlson 2019a “GO.Db: A Set of Annotation Maps Describing the Entire Gene Ontology.”) and the Bioconductor *D. melanogaster* annotation data package org.Dm.e.g.db (objects org. Dm.egGO2ALLEGS and org. Dm.egENSEMBL) (Carlson 2019b, “Org.Dm.Eg.Db: Genome Wide Annotation for Fly.”). We used the gene analysis mode to account for gene length heterogeneity among GO categories. Given the close proximity of genes in the *Drosophila* genome, we used the gene definition mode that does not search for SNPs in the up- and downstream flanking regions of each gene. To search for tissue enrichment, we substituted the Gowinda GO database with the Flyatlas2 tissue-specific expression profiles (Leader *et al.* 2018) and executed Gowinda in a similar manner as described above. Further information on the identified genes was acquired from www.flybase.org, http://flyatlas.gla.ac.uk/FlyAtlas2/index.html and www.uniprot.org (last accessed on 30-08-2021).

## Results and discussion

**Fig. 2.**
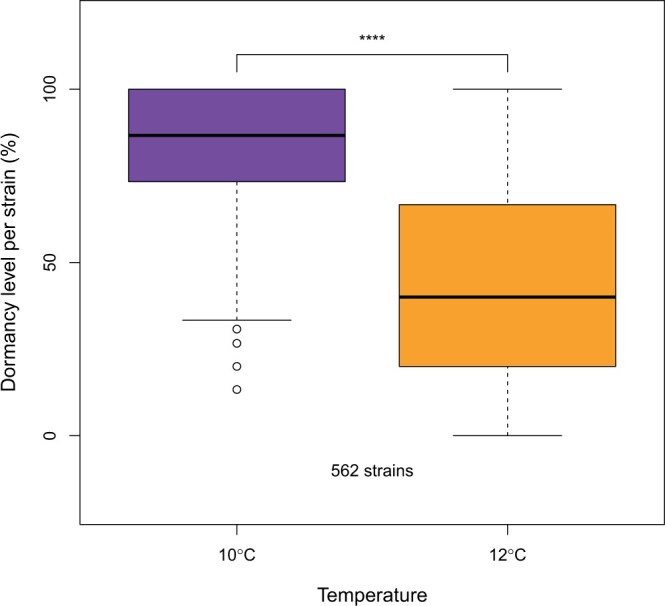
Dormancy expression at 2 temperature regimes (10 and 12°C, LD 10:14) of the South African *D. simulans* population (562 strains). The dormancy levels between the 2 temperatures were compared with the Wilcoxon signed-rank test. The decrease in dormancy from 12 to 10°C demonstrates the plastic character of the trait.

**Fig. 3.**
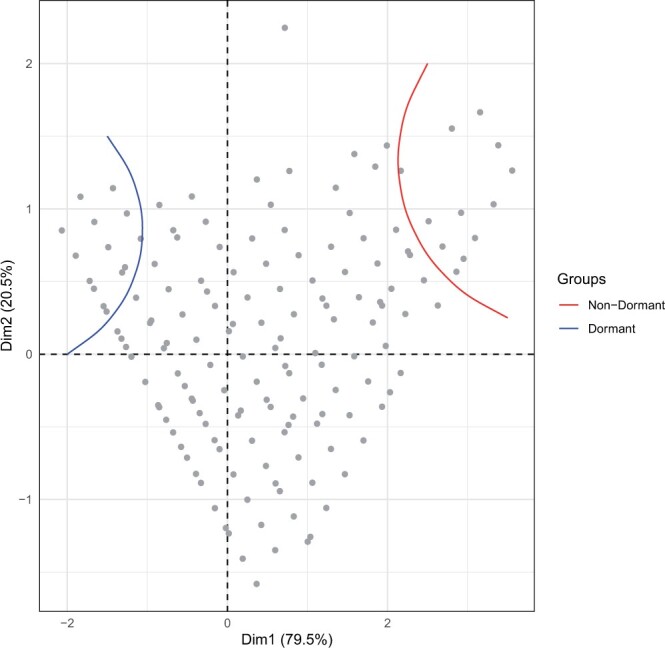
PC analysis of the dormancy levels of 562 *D. simulans* isofemale strains from South Africa at 2 temperature regimes (10 and 12°C, LD 10:14). Two out of 3 vertices of the triangular-shaped position of the strains harbor the most extreme nondormant and dormant strains. The third vertex includes the strains that showed the greatest difference in dormancy incidence between the 2 temperature regimes. Please note that some isofemale strains had identical levels of dormancy, thus they are superimposed in the figure.

A subset of 562 isofemale strains from the South African *D. simulans* population was screened for dormancy. As expected, given the plastic nature of the trait, dormancy levels were lower at 10°C compared to those from 12°C (Fig. 2, Supplementary Fig. 1 and Supplementary Table 1, Wilcoxon signed-rank test *P*-value < 2e-16). In conjunction with this, fewer eggs per fly were produced at 10°C (Supplementary Fig. 2, Wilcoxon signed-rank test *P*-value < 2e-16); 23.4% and 24.8% of observed variance is explained by differences between strains for the 10 and 12°C temperature regimes, respectively. A PCA on the dormancy levels of all strains (Fig. 3) resulted in a triangular shape where isofemale strains with extreme dormancy phenotypes were clustered at 2 out of 3 vertices (the third vertex includes the strains that showed the greatest difference in dormancy incidence between the 2 temperature regimes).

**Fig. 4.**
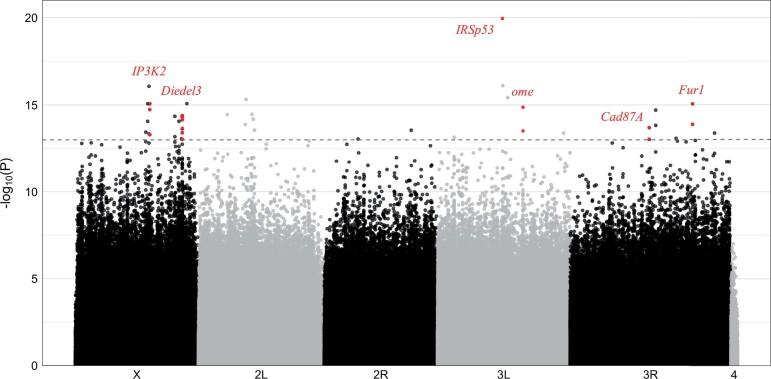
Manhattan plot of the adjusted chi-squared test *P*-values from the Pool-GWAS for dormancy. The dashed line indicates the significance threshold of 10^*−*13^. Genes that are discussed in the main text are highlighted in red.

We created replicate pools from the most extreme 25 strains for each extreme phenotype and performed Pool-Seq (library sizes ranged from 31,741,963 to 54,774,126). The merged replicates had an average overall coverage of 93 and an adjusted chi-squared test between the nondormant and dormant group was applied to ∼3.85 million SNPs (Fig. 4). A FDR correction according to Bastide *et al.* (2013) did not return any significant SNPs (Supplementary Fig. 3). However, this is not surprising because this method is extremely conservative. For a simple genetic architecture and/or large effect loci, such as in the case of female abdominal pigmentation, this procedure is sufficiently powerful. Hence we should have identified candidates if a small number of loci are contributing most of the variation in dormancy. On the other hand, for complex traits, the power can be too low to detect contributing loci with this conservative approach (Bastide *et al.* 2013). Consistent with a highly polygenic architecture, a PCA of the allele frequencies for polymorphic SNPs separated the 2 groups very well, irrespective of whether chromosomes or chromosome arms were used (Supplementary Figs. 4–9).

Reasoning that even for a complex trait like dormancy, some loci may contribute more to the phenotypic variation in the population than others (either by larger effect sizes or higher frequency), we followed a strategy widely used in *Drosophila* and scrutinized SNPs that do not pass a multiple testing correction, but had an uncorrected *P*-value smaller than an ad hoc chosen threshold of 1e-13 to define candidate SNPs for dormancy-related effects. Note that this threshold is lower than the lowest *P*-value (9.55e-13) obtained from permutations (randomly changing labels of replicates from both groups before merging), which implies that our threshold is not very liberal and the identified signals are likely to reflect a true biological signal.

Out of 43 candidate SNPs, most were located in UTR sequences and only 1 nonsynonymous substitution was detected (Supplementary Table 2). A chi-square goodness of fit test did not show an enrichment of candidate SNPs across chromosomes. The absolute log odds ratio of these candidate SNPs (Supplementary Table 2) was among the top 3.83% of absolute log odds ratios of all SNPs. No significant GO term or tissue enrichment was observed after multiple testing correction (Supplementary Tables 3 and 4). However, this is not surprising given the small number of genes identified (25) (Supplementary Table 2). Interestingly, previously identified candidate genes, such as *cpo*, were not significant, similar to a very powerful recently published GWAS in *D. melanogaster* from North America and the Caribbean (Erickson *et al.* 2020).

The strongest association was detected for the gene *IRSp53* (*Insulin receptor substrate 53 kDa*, *P*-value = 1.1e-20). *IRSp53* is highly expressed in the female fly eye and throughout its gastrointestinal system, and is differentially expressed in expression studies of dormancy and cold acclimation in *D. melanogaster* (Baker and Russell 2009; MacMillan *et al.* 2016; Zhao *et al.* 2016; Zare *et al.* 2018). Nevertheless, since only a single SNP in *IRSp53* showed this strong association, it may still be a false positive. For more confidence in candidate genes, we required at least 2 candidate SNPs per gene with *P*-values smaller than the significance threshold of 10^−13^ (Supplementary Table 2).

*Inositol 1,4,5-triphosphate kinase 2* (*IP3K2*; chromosome X) harbored 3 candidate SNPs in its 5′UTR (*P*-value ≥ 8.59e-16). It is regulated by ecdysteroids (Van Bortle *et al.* 2015) and participates in cold acclimation (MacMillan *et al.* 2016) and apoptotic/autophagic cell death (Terhzaz *et al.* 2010; Nelson *et al.* 2014) in *D. melanogaster*. Cell death is of particular interest, as this process is an integral part of the mid-oogenesis checkpoint that blocks oogenesis under dormancy-inducing conditions (Lirakis *et al.* 2018). Interestingly, inositol 1,4,5-triphosphate signaling regulates ovulation in *Caenorhabditis elegans* (Clandinin *et al.* 1998; Bui and Sternberg 2002). On the same chromosome, the gene *Diedel3*, which is surrounded by many candidate SNPs (*P*-value ≥ 4.16e-15), is highly expressed in the midgut and associated to insulin signaling in *D. melanogaster* (Palu and Thummel 2016). On chromosome 3, we identified *omega* (*ome*, *P*-value ≥ 1.38e-15) that encodes a dipeptidyl-peptidase and is also highly expressed in the midgut, *Cadherin 87A* (*Cad87A*, *P*-value ≥ 2.01e-14) that is involved in calcium-dependent cell–cell adhesion and is a juvenile hormone-induced gene (Li *et al.* 2007), and *Furin 1* (*Fur1*, *P*-value ≥ 9.10e-16) that exhibits serine-type endopeptidase activity. Interestingly, both *ome* and *Cad87A* function in the ovary (Chihara *et al.* 2005; Zartman *et al.* 2009). *Cad87A* exhibits latitudinal differential expression in male *D. melanogaster*, possibly indicative of spatially varying selection (L. Zhao *et al.* 2015). *Fur1* harbors 2 SNPs of particularly high effect. Although we did not find a link between *Fur1* and the dormancy expression machinery in *Drosophila*, its homologous gene in *C. elegans*, *kpc-1*, participates in dauer diapause formation (Schroeder *et al.* 2013; Hung *et al.* 2014).

We further searched for structural polymorphisms by altering the filtering criteria in our pipeline and including several regions with high coverage (Supplementary File 3). This alternative analysis unraveled 2 regions with large differences in coverage between the 2 dormancy groups (up to 101 difference in coverage) and very low *P*-values (< 10^−13^): 1 region in chromosome X and 1 region in chromosome 2R (Supplementary Fig. 10 and Supplementary Table 5). Sequencing single flies from 12 strains of the nondormant group identified coverage heterogeneity among individuals in these regions. In fact, only a single fly (strain SS1294) had high coverage (Supplementary Figs. 11 and 12). For this reason, we did not further pursue structural variation as a major contributor to dormancy variation. Beyond the present study, these results have important implications for Pool-GWAS studies. While Pool-GWAS provides a cost-effective alternative to classic GWAS with individual sequencing, in particular for large sample sizes (Schlötterer *et al.* 2014), we demonstrated that it may not be the most suitable method to study the contribution of structural polymorphism to phenotypic variation.

To conclude, dormancy, either in the form of diapause or quiescence (Koštál 2006), is a complex trait that mobilizes many molecular pathways during its expression. Similar to other cold-related traits (MacKay *et al.* 2012; Freda *et al.* 2017; Teets and Hahn 2018; Lecheta *et al.* 2020), diapause/quiescence are expected to have a polygenic basis (Ragland *et al.* 2019). Our analysis confirmed that diapause in *D. simulans* is a complex trait with many contributing loci, each of small effect. Even the SNPs that showed a significant association only showed a modest difference in allele frequency between high and low dormancy flies, indicating that even the most significant loci have only very moderate effects.

We caution that for polygenic traits, the genetic architecture differs due to frequency differences of contributing loci as frequently evidenced by the poor transfer of polygenic scores across populations (Mathieson 2021). Consistent with this, QTL mapping identified different sets of contributing loci in different populations (Conte *et al.* 2015; Swarts *et al.* 2021) and replicate populations in experimental evolution studies adapted to the same selection pressure using alternative sets of genes (Griffin *et al.* 2017; Barghi *et al.* 2019). Thus, it is not surprising that our study did not find associations with previously identified candidate genes in *Drosophila*.

It remains nevertheless an open question why the previously identified candidate gene *cpo* in populations from the US East Coast (Schmidt *et al.* 2008; Cogni *et al.* 2014) was not detected in a GWAS for dormancy in *D. melanogaster* from North America and the Caribbean (Erickson *et al.* 2020). It may be possible that the causative alleles of *cpo* were at too low frequencies to be detected. Alternatively, the effect of *cpo* may have arisen from the association of multiple small effect alleles with opposing effects on segregating inversions, as suggested by the enrichment of seasonal SNPs in chromosomal inversions (Machado *et al.* 2021).

Finally, in the present study, dormancy phenotyping was strictly oogenesis-oriented. As a result, associations to other features of dormancy may have been missed and any identified association may be specifically linked to oogenesis (rather than dormancy in general). Despite this limitation, our Pool-GWAS on reproductive dormancy in *D*. *simulans* identified several candidate genes and functional conservation in *C. elegans* further strengthens our results. We foresee that the genes identified here will be targets for future dormancy studies.

## Data availability

The sequencing data underlying this article are available in the European Nucleotide Archive (ENA) at https://www.ebi.ac.uk/ena/browser/view/ and can be accessed with the Primary Accession code PRJEB37936. The phenotyping data are included in Supplementary Table 1.

Supplemental material is available at *G3* online.

## Supplementary Material

jkac027_Supplementary_Figure_S1

jkac027_Supplementary_Figure_S2

jkac027_Supplementary_Figure_S3

jkac027_Supplementary_Figure_S4

jkac027_Supplementary_Figure_S5

jkac027_Supplementary_Figure_S6

jkac027_Supplementary_Figure_S7

jkac027_Supplementary_Figure_S8

jkac027_Supplementary_Figure_S9

jkac027_Supplementary_Figure_S10

jkac027_Supplementary_Figure_S11

jkac027_Supplementary_Figure_S12

jkac027_Supplementary_File_2

jkac027_Supplementary_File_3

jkac027_Supplementary_Tables

jkac027_Supplementary_Data
