# Supplementary material for: Pool-GWAS on reproductive dormancy in Drosophila simulans suggests a polygenic architecture
Source: G3 (Bethesda). 2022 Feb 7;12(3):jkac027. doi: 10.1093/g3journal/jkac027 (PMC8895979; doi:10.1093/g3journal/jkac027)
Supplement: jkac027_Supplementary_Figure_S1 [file jkac027_supplementary_figure_s1.pdf]

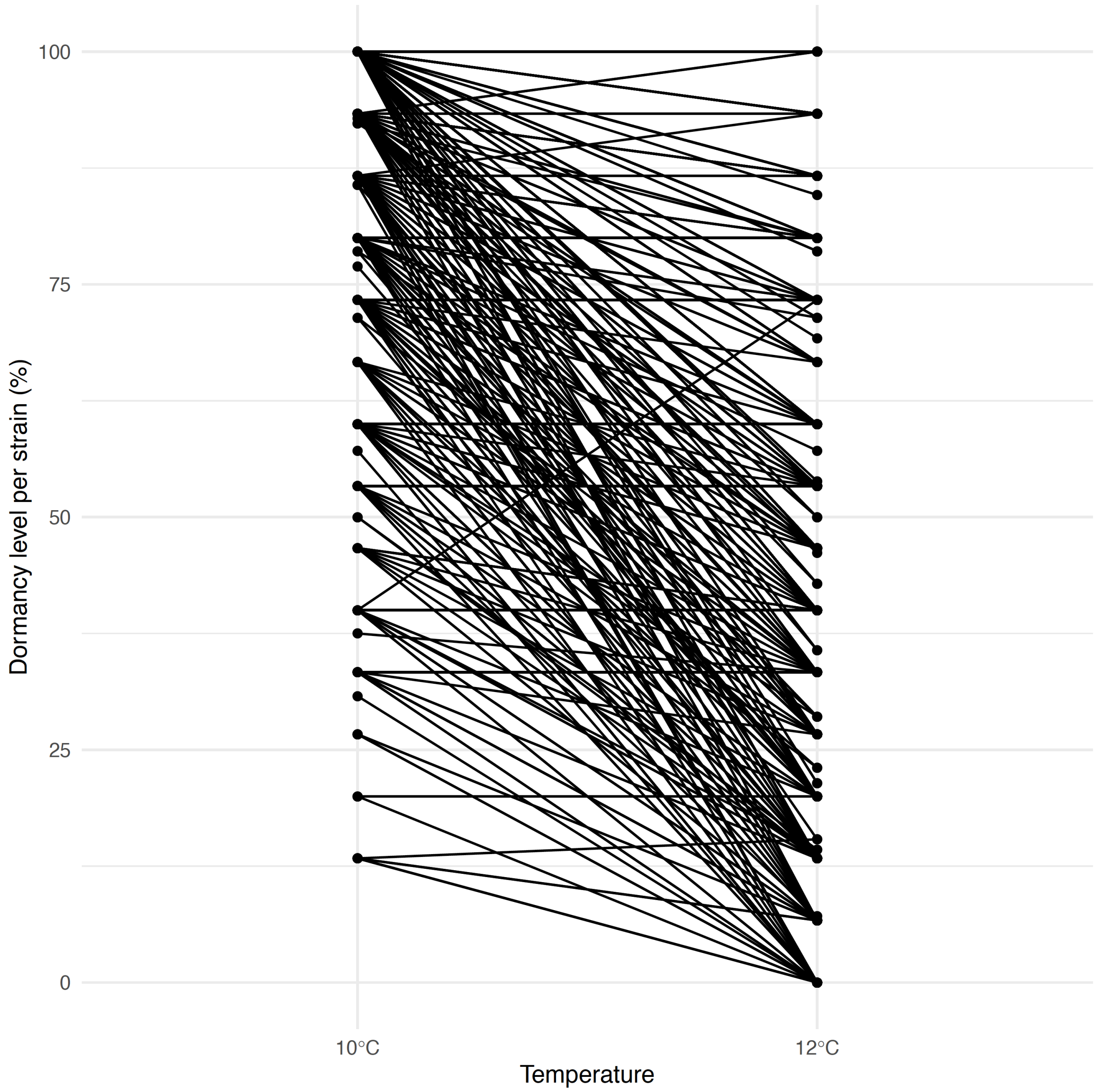


**Figure S1:** Dormancy expression at two temperature regimes (10°C and 12°C, LD 10:14) of the South African *D. simulans* population (562 strains). The decrease in dormancy from 12°C to 10°C demonstrates the plastic character of the trait.
