# Supplementary material for: Pool-GWAS on reproductive dormancy in Drosophila simulans suggests a polygenic architecture
Source: G3 (Bethesda). 2022 Feb 7;12(3):jkac027. doi: 10.1093/g3journal/jkac027 (PMC8895979; doi:10.1093/g3journal/jkac027)
Supplement: jkac027_Supplementary_Figure_S2 [file jkac027_supplementary_figure_s2.pdf]

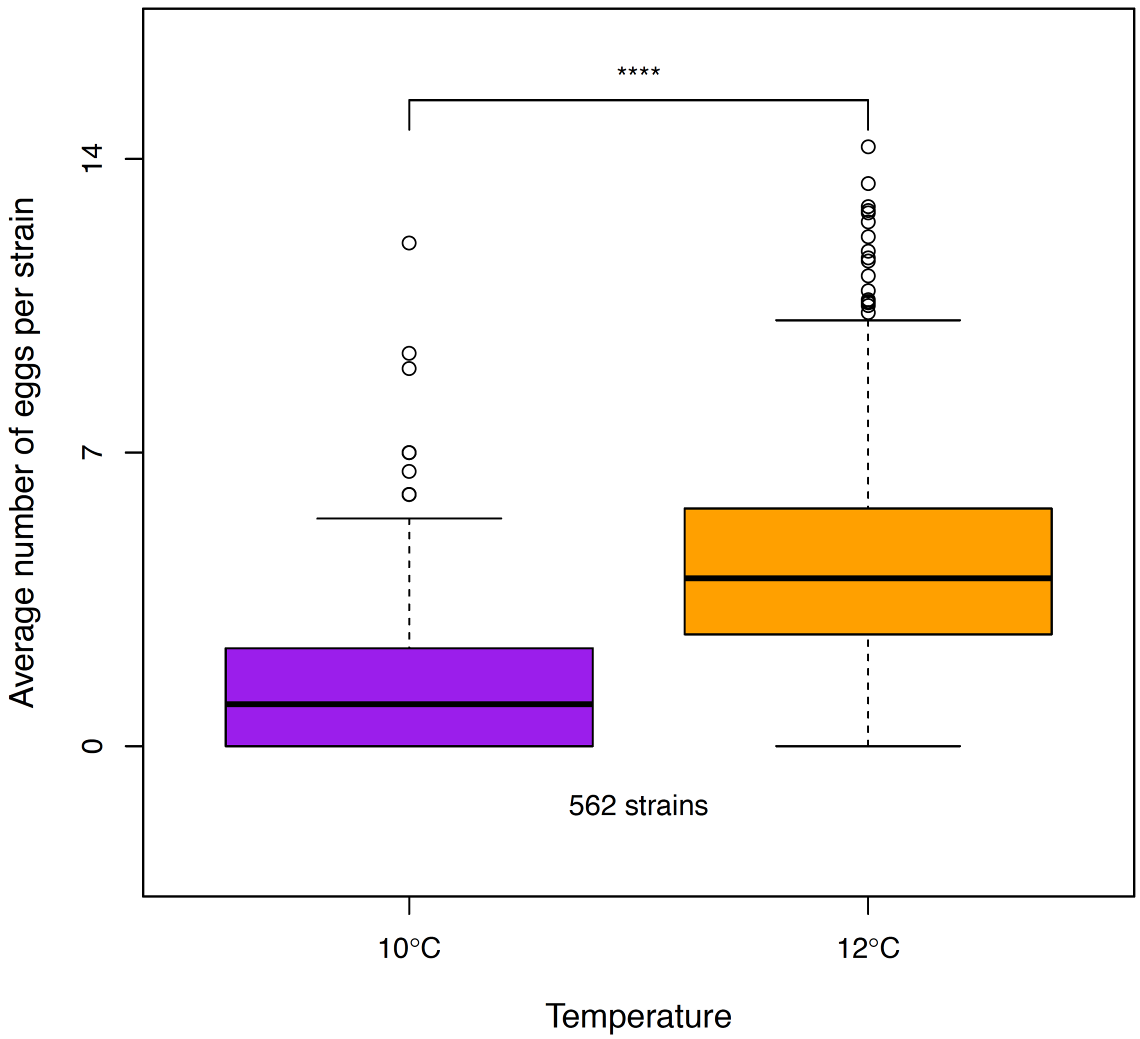

Figure S2: Average number of eggs produced per strain (562 strains of the South African *D. simulans* population) at two temperature regimes (10°C and 12°C, LD 10:14). The average number of eggs between the two temperatures were compared with the Wilcoxon signed-rank test. Only the flies that produced eggs were used to calculate these values.
