# Supplementary material for: Pool-GWAS on reproductive dormancy in Drosophila simulans suggests a polygenic architecture
Source: G3 (Bethesda). 2022 Feb 7;12(3):jkac027. doi: 10.1093/g3journal/jkac027 (PMC8895979; doi:10.1093/g3journal/jkac027)
Supplement: jkac027_Supplementary_Figure_S3 [file jkac027_supplementary_figure_s3.pdf]

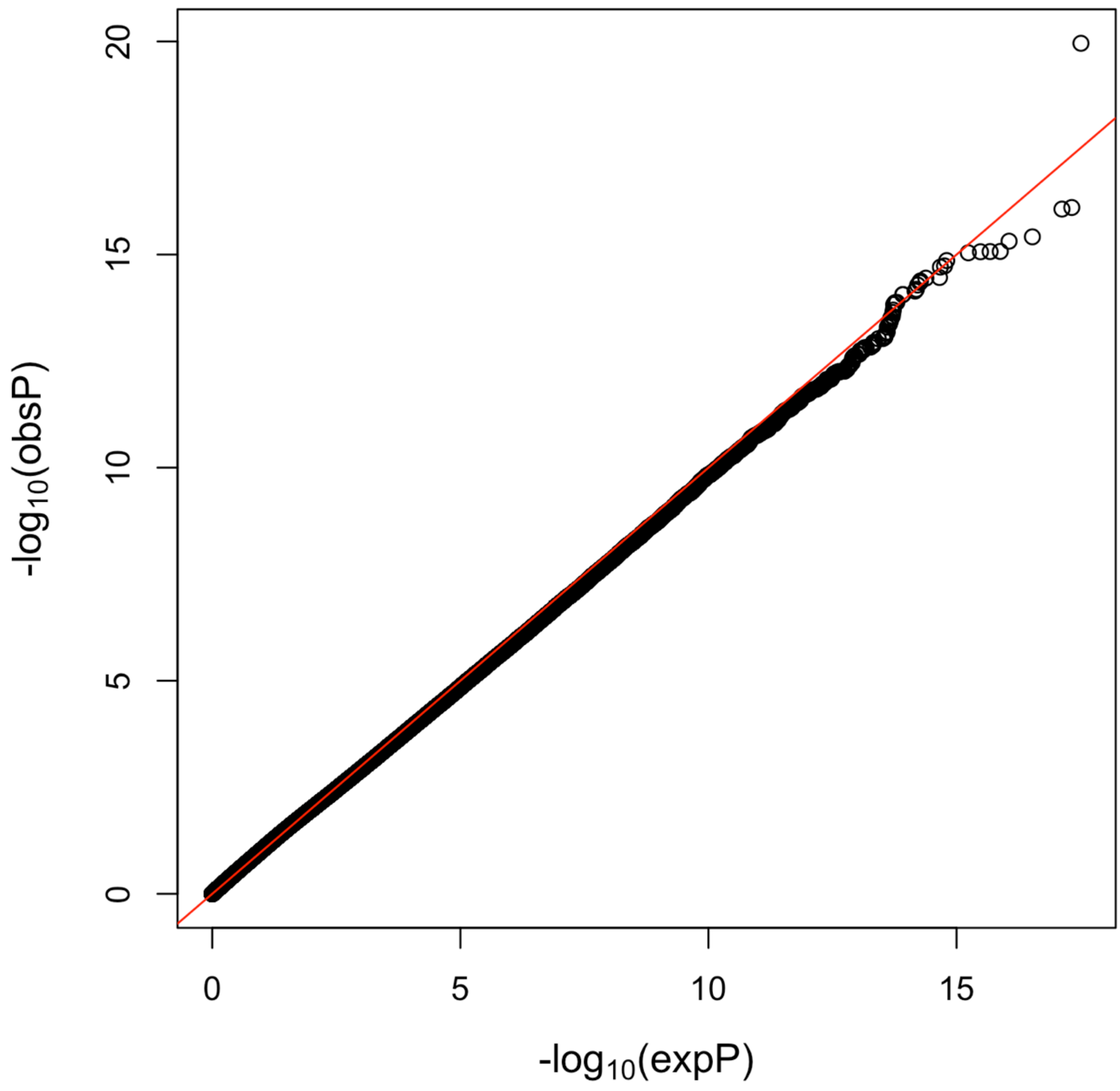

Figure S3: Q-Q plot for observed *p*-values from the Pool-GWAS and the simulated distribution obtained under the null (using an *a* value of 0.8, see Suppl. File 2). The plot shows the –log10 *p*-values.
