# Supplementary figures and images for: Pool-GWAS on reproductive dormancy in Drosophila simulans suggests a polygenic architecture
Source: G3 (Bethesda). 2022 Feb 7;12(3):jkac027. doi: 10.1093/g3journal/jkac027 (PMC8895979; doi:10.1093/g3journal/jkac027)

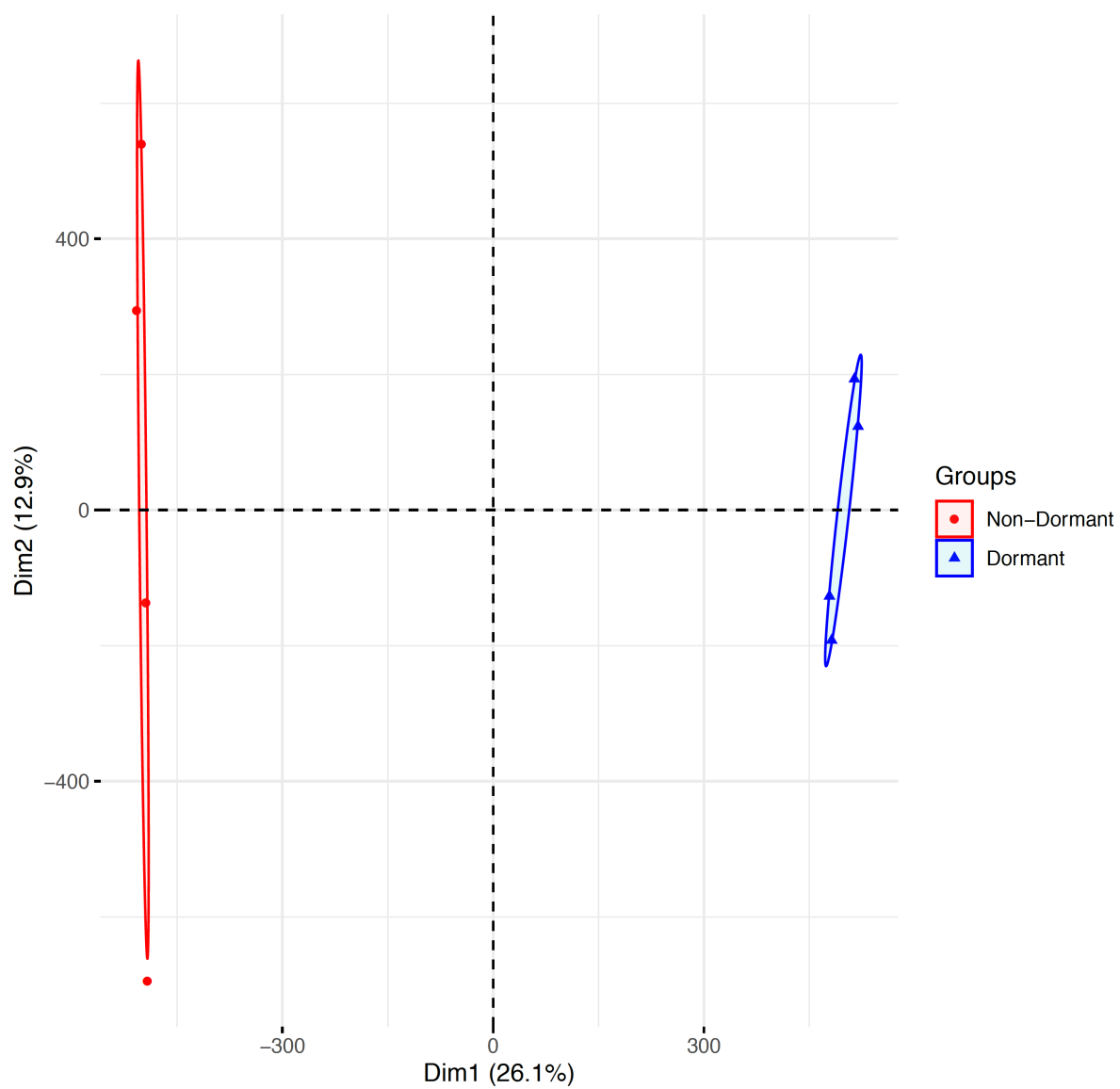

**Figure S4:** PCA of the allele frequencies for polymorphic SNPs on chromosome X.

Supplement: jkac027_Supplementary_Figure_S4 [file jkac027_supplementary_figure_s4.pdf]

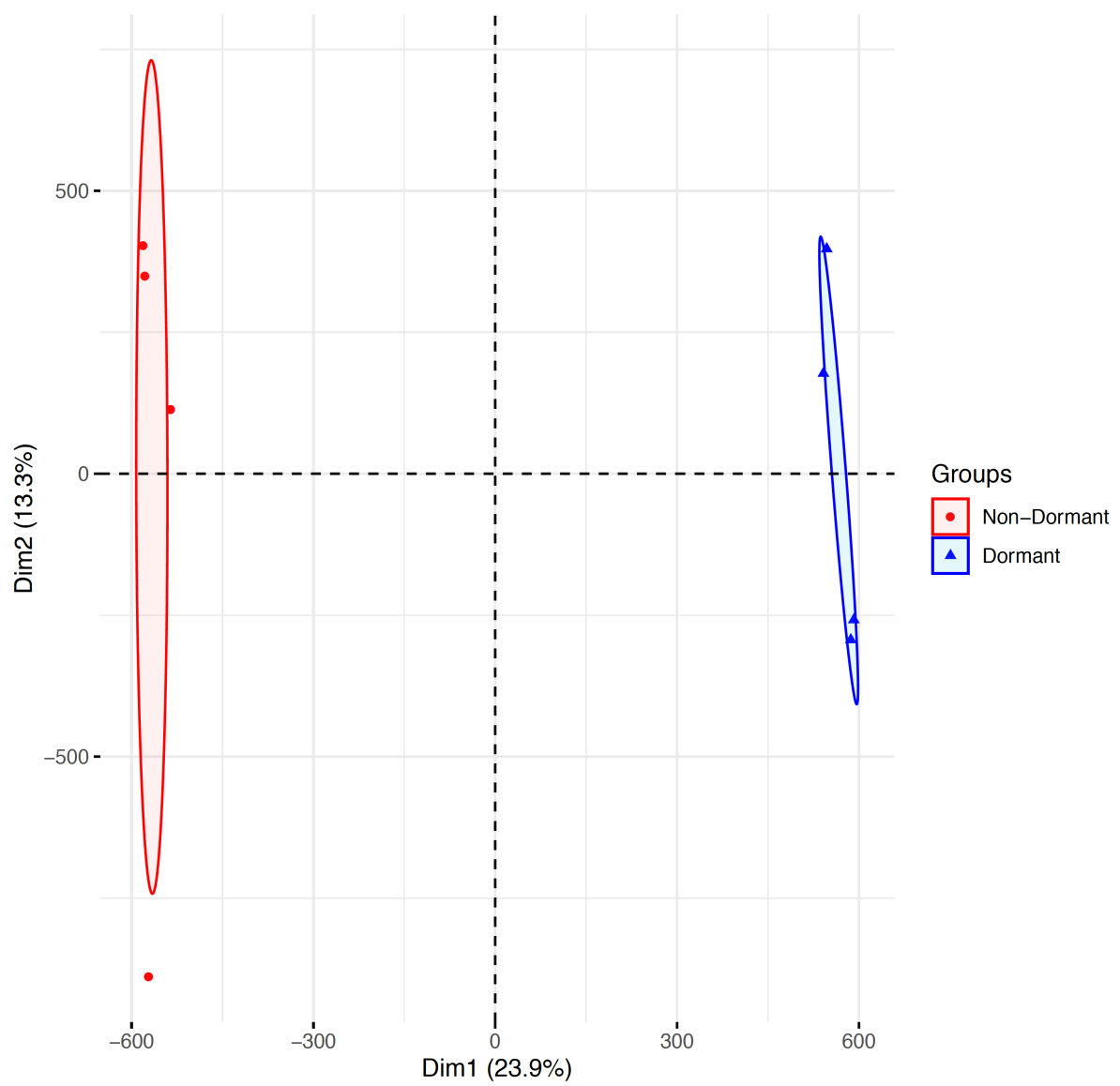

**Figure S5:** PCA of the allele frequencies for polymorphic SNPs on chromosome 2L.

Supplement: jkac027_Supplementary_Figure_S5 [file jkac027_supplementary_figure_s5.pdf]

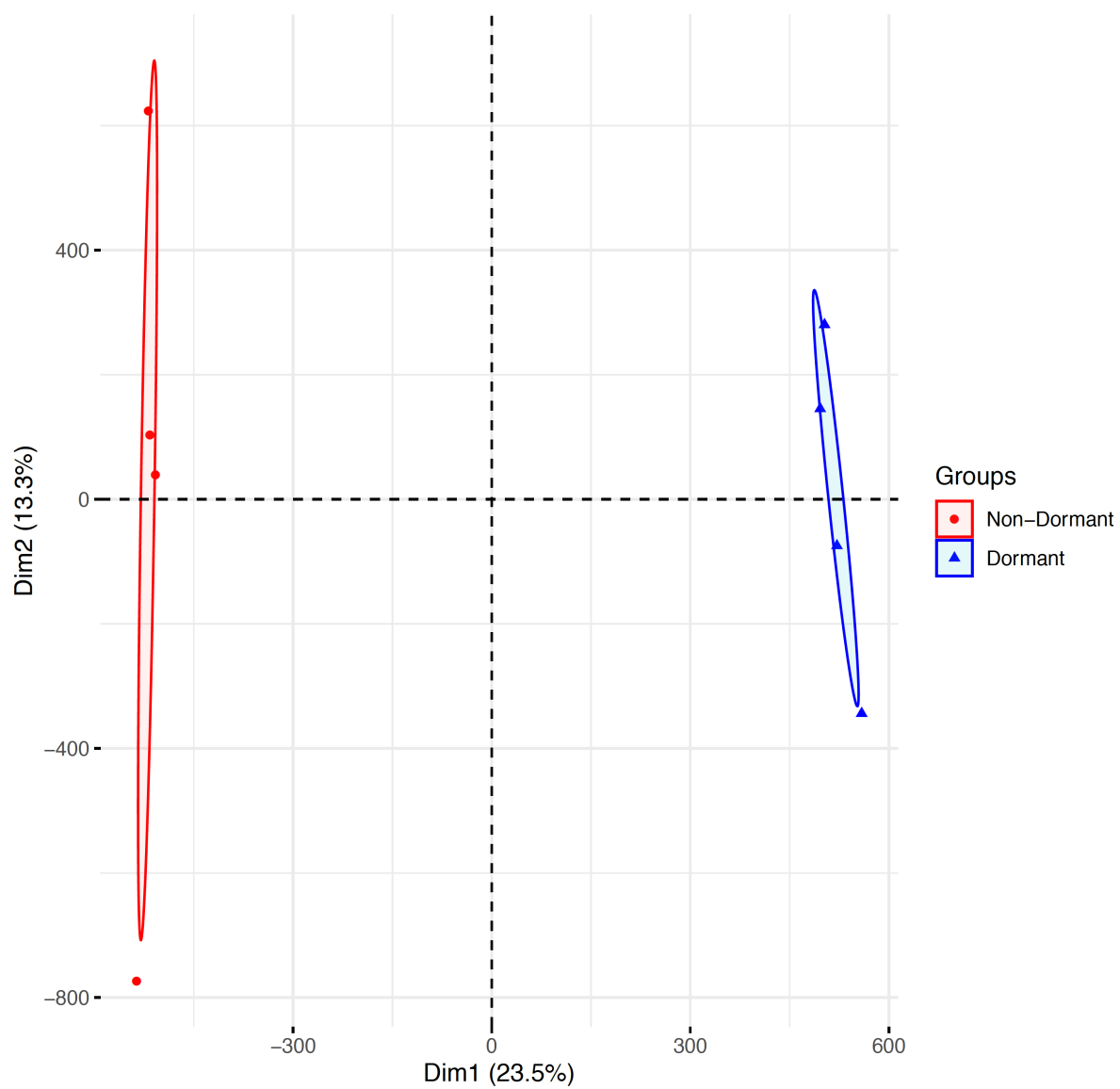

**Figure S6:** PCA of the allele frequencies for polymorphic SNPs on chromosome 2R.

Supplement: jkac027_Supplementary_Figure_S6 [file jkac027_supplementary_figure_s6.pdf]

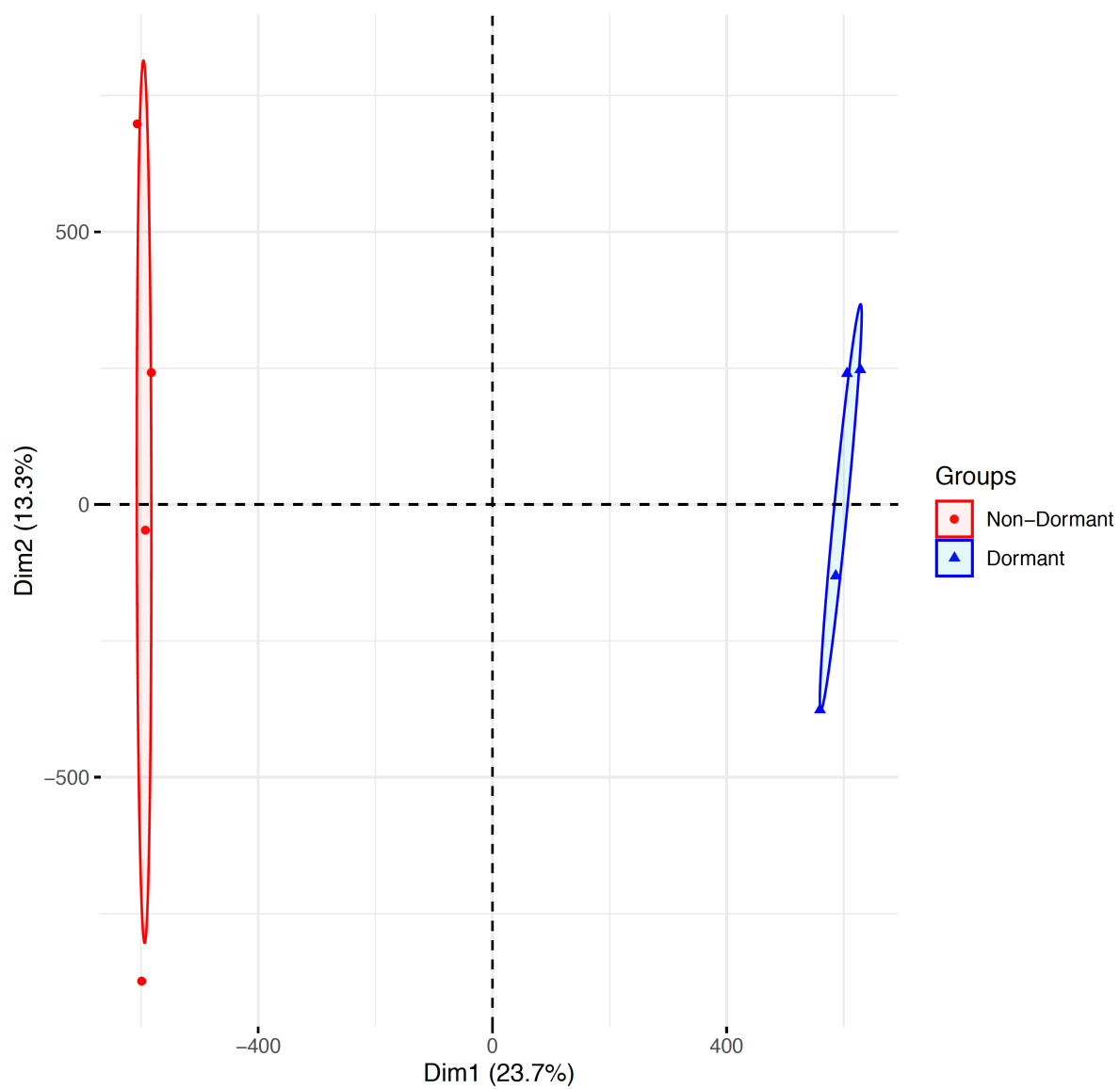

**Figure S7:** PCA of the allele frequencies for polymorphic SNPs on chromosome 3L.

Supplement: jkac027_Supplementary_Figure_S7 [file jkac027_supplementary_figure_s7.pdf]

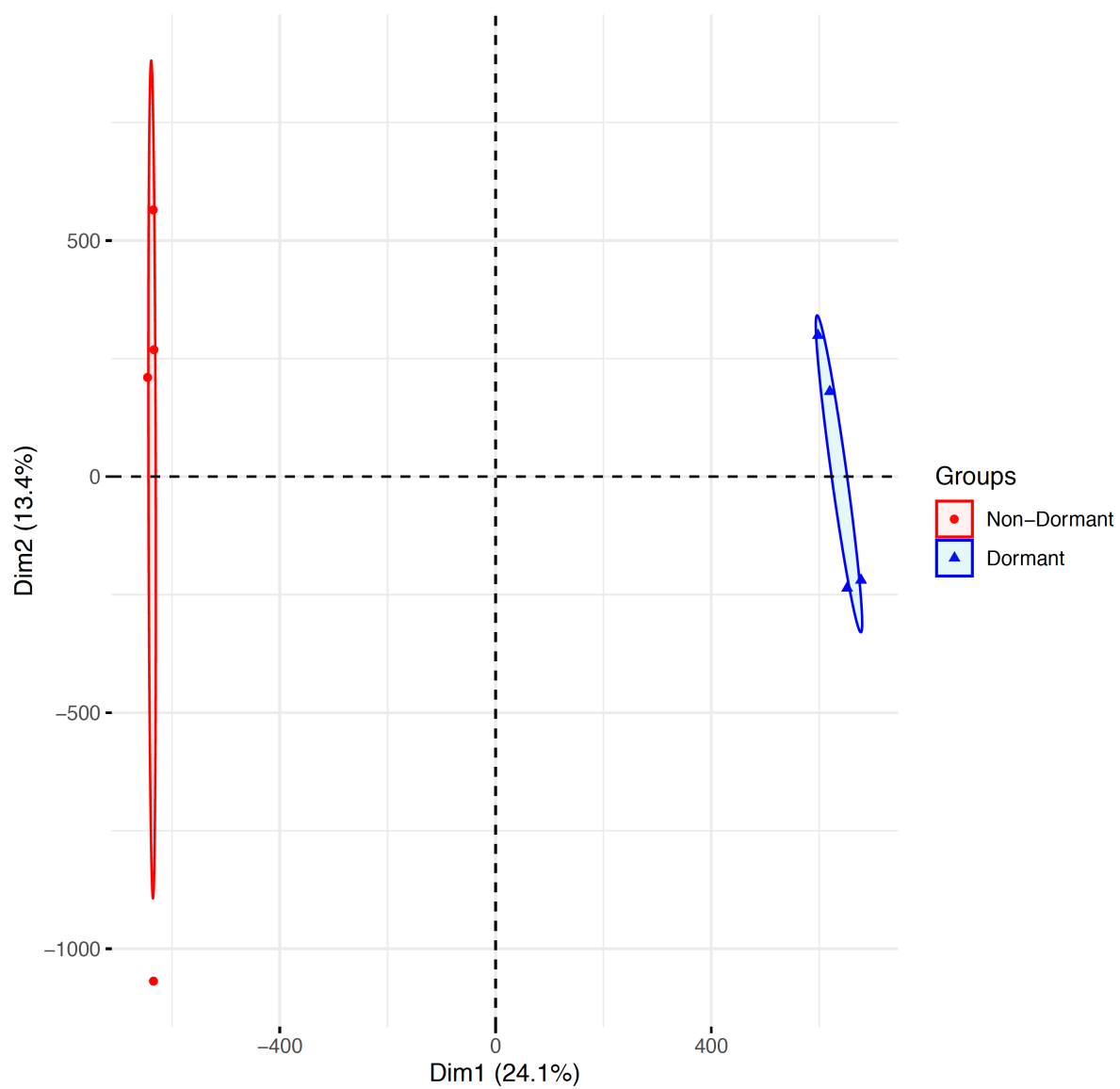

**Figure S8:** PCA of the allele frequencies for polymorphic SNPs on chromosome 3R.

Supplement: jkac027_Supplementary_Figure_S8 [file jkac027_supplementary_figure_s8.pdf]

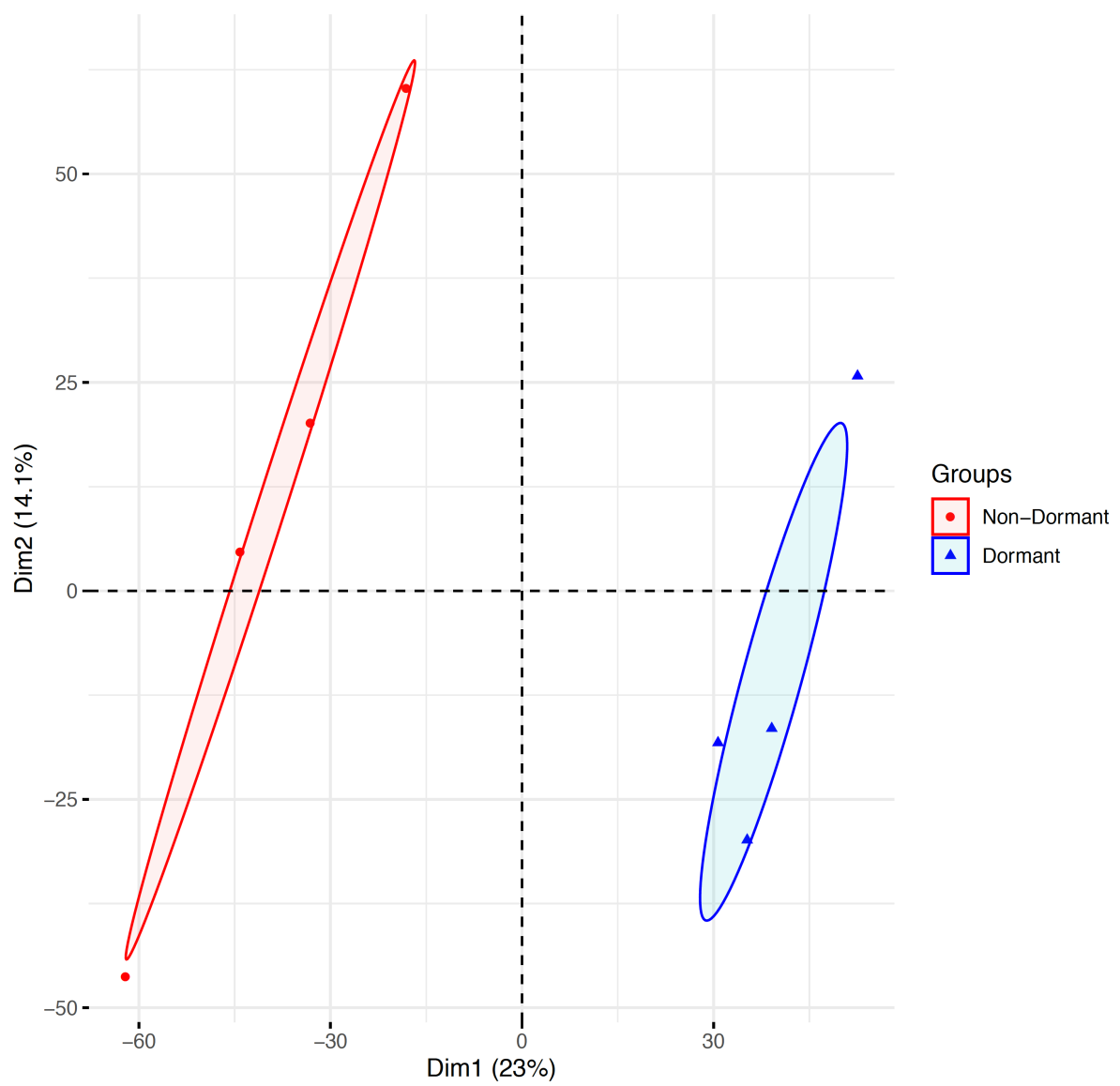

**Figure S9:** PCA of the allele frequencies for polymorphic SNPs on chromosome 4.

Supplement: jkac027_Supplementary_Figure_S9 [file jkac027_supplementary_figure_s9.pdf]
