# Supplementary material for: Pool-GWAS on reproductive dormancy in Drosophila simulans suggests a polygenic architecture
Source: G3 (Bethesda). 2022 Feb 7;12(3):jkac027. doi: 10.1093/g3journal/jkac027 (PMC8895979; doi:10.1093/g3journal/jkac027)
Supplement: jkac027_Supplementary_Figure_S10 [file jkac027_supplementary_figure_s10.pdf]

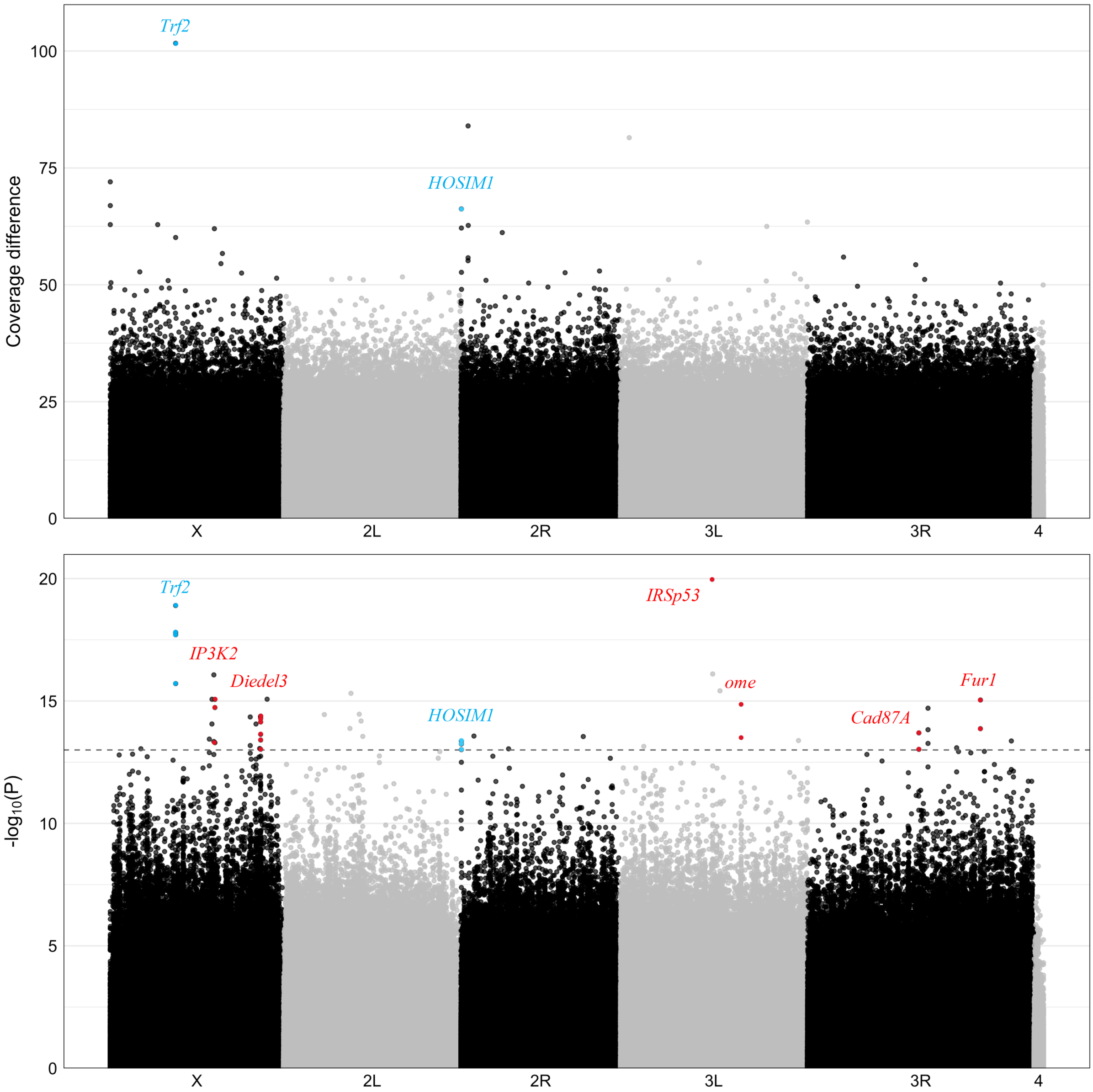

Figure S10: Coverage differences between the Non-Dormant and Dormant group, using the dataset that includes regions of high coverage (top) and manhattan plot of the adjusted chi-squared test *p*-values from the Pool-GWAS for dormancy, using the same dataset (bottom). The dashed line indicates the arbitrary threshold of 10^{-13}. The plot is annotated for genes that were previously identified (red) and the two newly unraveled regions with high coverage (blue).
