# Supplementary material for: Pool-GWAS on reproductive dormancy in Drosophila simulans suggests a polygenic architecture
Source: G3 (Bethesda). 2022 Feb 7;12(3):jkac027. doi: 10.1093/g3journal/jkac027 (PMC8895979; doi:10.1093/g3journal/jkac027)
Supplement: jkac027_Supplementary_Figure_S11 [file jkac027_supplementary_figure_s11.pdf]

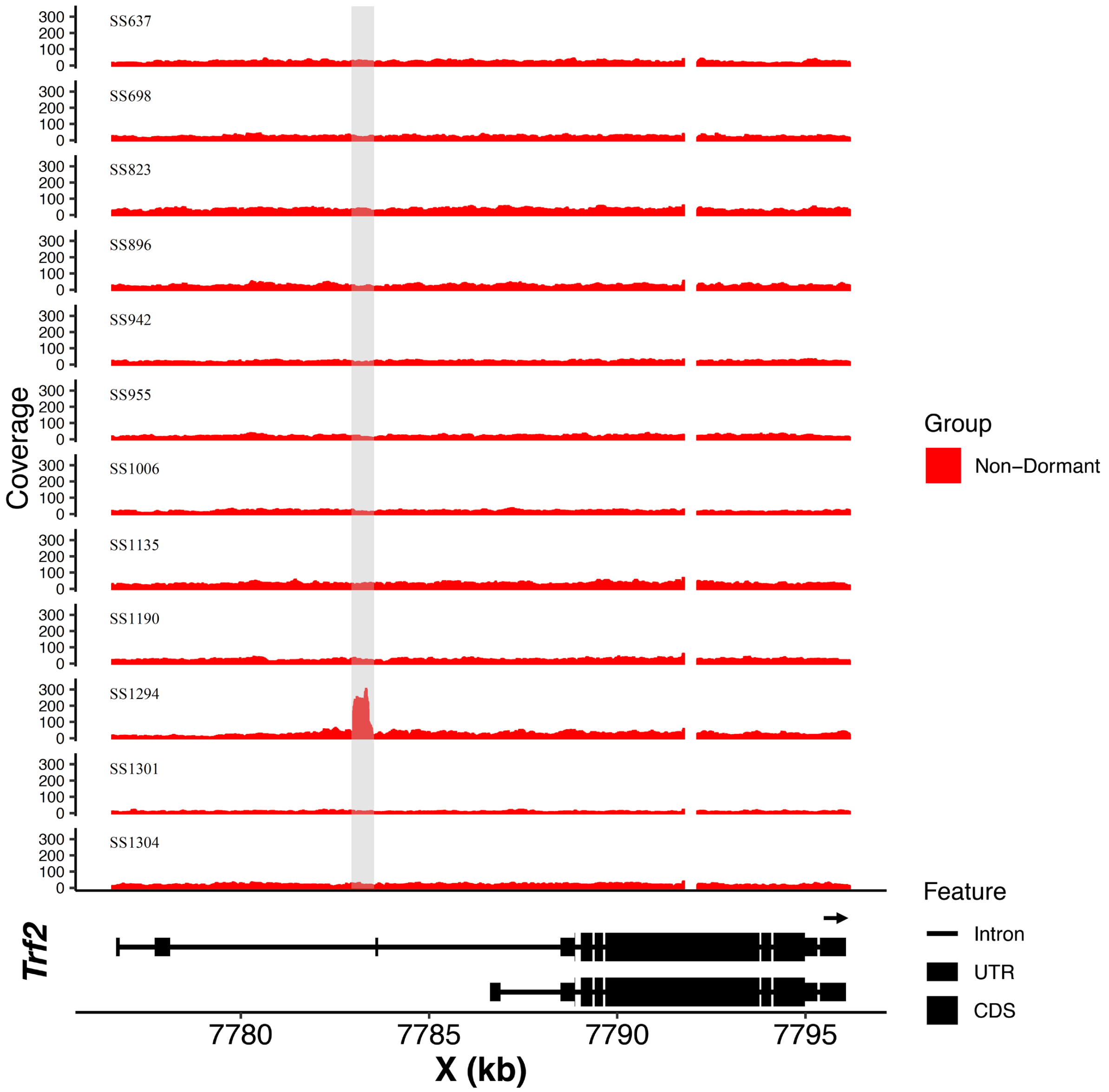

Figure S11: Coverage of the *Trf2* region on chromosome X for 12 individually sequenced Non-Dormant strains. Only one strain, SS1294, exhibits high coverage at the duplication breakpoint region of *Trf2*. This duplication creates the Paris *Sex-Ratio* Drive.
