# Supplementary material for: Pool-GWAS on reproductive dormancy in Drosophila simulans suggests a polygenic architecture
Source: G3 (Bethesda). 2022 Feb 7;12(3):jkac027. doi: 10.1093/g3journal/jkac027 (PMC8895979; doi:10.1093/g3journal/jkac027)
Supplement: jkac027_Supplementary_Figure_S12 [file jkac027_supplementary_figure_s12.pdf]

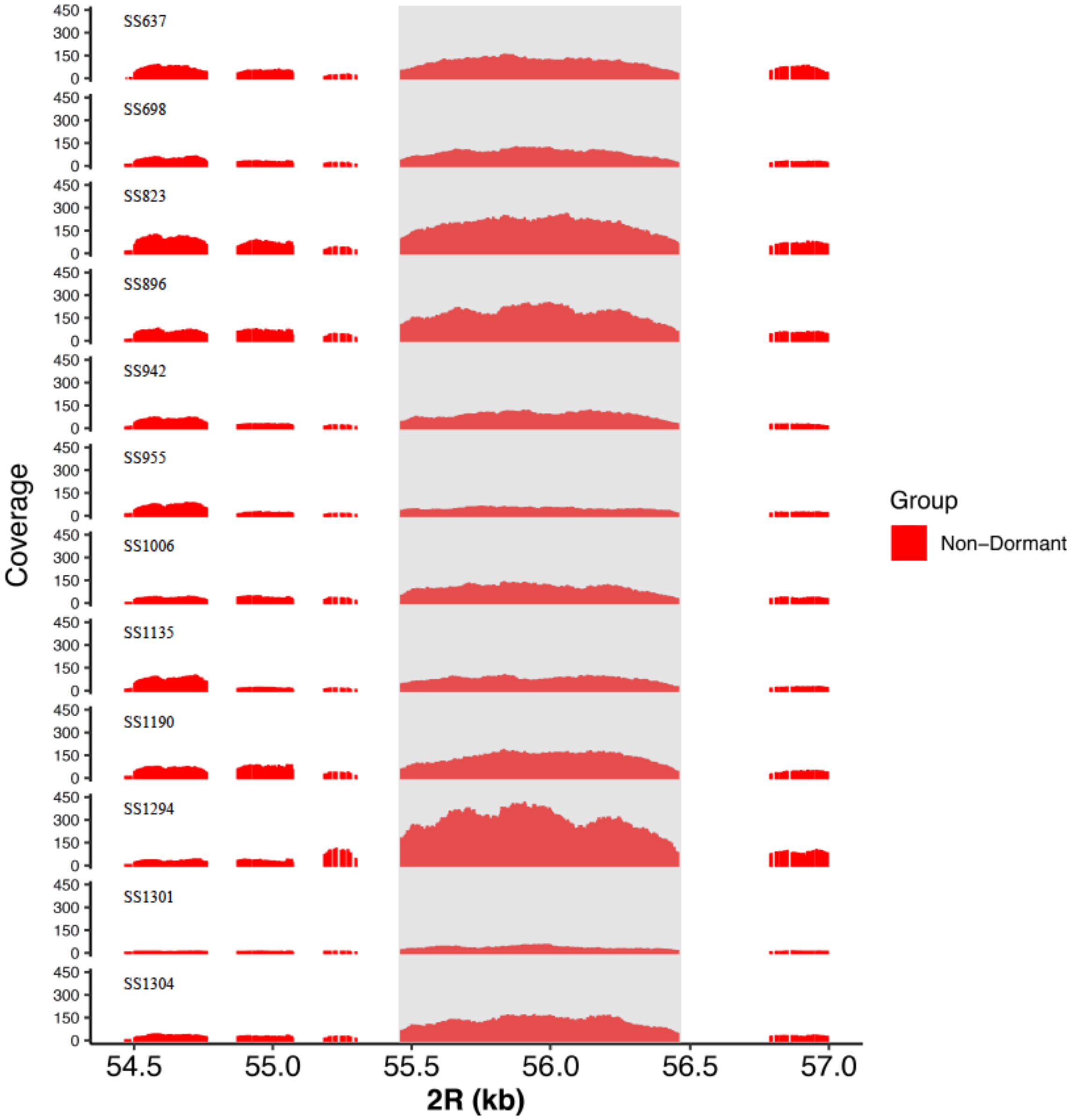

Figure S12: Coverage of the *HOSIM1* region in the beginning of 2R for 12 individually sequenced Non-Dormant strains. The strain SS1294, which is the only one exhibiting high coverage at the duplication breakpoint region of *Trf2*, exhibits the highest coverage in this 2R region.
