# Supplementary material for: Pool-GWAS on reproductive dormancy in Drosophila simulans suggests a polygenic architecture
Source: G3 (Bethesda). 2022 Feb 7;12(3):jkac027. doi: 10.1093/g3journal/jkac027 (PMC8895979; doi:10.1093/g3journal/jkac027)
Supplement: jkac027_Supplementary_File_2 [file jkac027_supplementary_file_2.pdf]

Library preparation and pool sequencing

Each replicate pool was homogenized at maximum speed in a 1600 Mini-G Automated Tissue Homogenizer and Cell Lyser (SPEX SamplePrep, Metuchen, NJ) to ensure complete disruption of each fly. Genomic DNA was extracted using a salting out protocol (Miller et al. 1988) with RNase A treatment and fragmented with a Covaris S2 (Covaris, Inc. Woburn, MA, USA). For each replicate pool, 100 ng DNA were used to prepare paired-end libraries with a target insert size of 270 bp using the NEBNext Ultra DNA II Library Prep Kit (New England Biolabs, Ipswich, MA). Dual index barcodes were introduced with 6 PCR cycles. After final double purification on AMPure XP beads (Beckman Coulter, Carlsbad, CA), the libraries were combined with additional samples from other experiments to fill one lane for 2 x 150 bp sequencing on a HiSeq X Ten (Table S6).

Sequencing data processing

Raw sequencing reads were trimmed with Readtools (1.4.1-6-g2e2ad2c-SNAPSHOT) (Gómez-Sánchez and Schlötterer 2018). Only reads with at least 50 bp and minimum average quality for the modified Mott algorithm at least equal to 18 were kept. Trimmed reads were mapped by novoalign (Version 3.03.02, parameters: -i 270,75 -F STDFQ -o SAM -r RANDOM) (http://www.novocraft.com/products/novoalign/) to the M252 reference genome (Palmieri et al. 2015) that included the reference mitochondrial sequence, two *Wolbachia pipientis* genomes (wMel_AE017196.1, wRi_CP001391.1), *Acetobacter pasteurianus* (AP011170.1) and *Lactobacillus brevis* (CP000416.1) genomes. To optimize the mapping parameters, we calculated the insert size descriptive statistics required by novoalign with picard (Version 2.1.0, tool CollectInsertSizeMetrics) (http://picard.sourceforge.net) and re-mapped the reads (final novoalign parameters: -i 230,52 -F STDFQ -o SAM -r RANDOM).

Mapped reads were sorted by query name prior to demultiplexing and by coordinate after demultiplexing using picard (tool SortSam). Bam files were demultiplexed by Readtools (program AssignReadGroupByBarcode), allowing at most 2 mismatches at the barcode alignment. Duplicate reads were removed by picard, reads with low mapping quality and improper pairing were removed by samtools (parameters: -q 20 -f 0x0002 -F 0x0004 –F 0x0008) (Version 1.3) (Li et al. 2009) and overlapping pairs of reads were trimmed with

bamUtil (Version 1.0.13) (https://github.com/statgen/bamUtil). The final bam files were converted to an mpileup file using samtools and then to a synchronized pileup file using PoPoolation2 (parameters: --min-qual 20) (Version 1.201) (Kofler et al. 2011). RepeatMasker (with the RMblast search engine) (Smit et al. 2013-2015) was used to create repeat (custom *Drosophila* database, parameters: -a -xsmall -gccalc -pa 4 -nolow -species drosophila) and TE (Supplementary File 1) (Quesneville et al. 2005) (parameters: -a -xsmall -gccalc -pa 4 -nolow) annotations. Subsequently, we used PoPoolation2 and these annotations to identify and remove the regions with repeats and TEs from the synchronized pileup file (filter-sync-by-gtf.pl). Using all polymorphic positions and the same R functions as described in the manuscript, we performed a Principal Component Analysis for each major chromosome or chromosomal arm and the mitochondrial DNA separately.

FDR correction (adjusted from Bastide et al. 2013)

To correct for multiple tests in the dormancy Pool-GWAS, we estimated the rate of false discoveries for a given *p*-value, i.e., the FDR. To obtain the FDR, we first estimated the distribution of the *p*-values from the CMH test under the null hypothesis. Under the null, there are two sources of sampling variation. The first source is the sampling of alleles between phenotypes; under the null model, the *n* copies of an allele assigned to the non-dormant or dormant group will be binomially distributed with parameters *n* and *p*, with:

$$p = \mathrm{P}(\text{group} \mid \text{allele})$$

the conditional probability of assigning an allele to the non-dormant or dormant group. We can therefore model this source of variation with a simple binomial model.

The second source of sampling variation occurs if there is a bias for or against sampling an allele in one of the groups, as might happen if an allele is over- or under-represented in a group due to population structure or technical issues (e.g., over- or under- amplification of an allele during the PCR step). That is, unlike in the simple binomial model, there might be random background variation in P(group | allele). We can account for this second source of sampling variation by expanding the binomial model above, using a beta-binomial model. In this approach, the P(group | allele) has the same mean value as under the simple binomial

model, but a non-zero variance. As suggested by the name of the beta-binomial model, the variation in P(group | allele) is modeled by drawing values for this parameter from a beta distribution, a flexible two parameter distribution (parameters α and β), with the mean equal to:

$$\alpha/(\alpha+\beta)$$

and a variance that decreases with increasing α and β:

$$(\alpha\beta)/[(\alpha+\beta)^2(\alpha+\beta+1)]$$

In the case where α and β become very large, the variance tends toward 0 and the beta-binomial model collapses to the simple binomial model.

Here, where the coverages are very close to equal between the non-dormant and dormant groups, the mean of P(group | allele) should be 0.5 under the null, so we use a beta-binomial with $\alpha = \beta$, giving a mean for the beta of 0.5 and a variance of $1/(8\alpha + 4)$ (note, however, that the ratio of these two parameters can be adjusted to account for unequal coverages). A priori, we don't know what variance to use, but we can change this α to optimize the fit of the beta-binomial model to the real data.

To test different values of α and ultimately to perform the FDR correction, we simulated data sets according to the beta-binomial model, allowing us to obtain a distribution of *p*-values under the null hypothesis that we can compare to the observed distribution of *p*-values. Each data set was simulated using a single value of α for the whole data set, α_{null}. Each simulated data set contains the same number of SNPs as in the real data (~3.85 million SNPs), with each SNP represented by a random 2 x 2 table (as there are 2 groups and 2 alleles). The coverages and allele frequencies for each SNP are taken from the data, as described below (i.e., the column sums and overall table sums of the simulated tables correspond to those from the data).

For each SNP, the random tables were generated using the following procedure:

(i) For a single SNP, we generated a random 2 x 2 table, with the two nominal variables being the group (non-dormant vs dormant) and allelic state (A_1 vs A_2).

(ii) To obtain the two values in the table for the A_1 allele, we distributed the n_{A1} copies of this allele randomly over the two groups (with n_{A1} obtained from the data). We assigned k of the n_{A1} copies to the non-dormant group by drawing a random k from a binomial distribution. This binomial has parameters $n = n_{A1}$ and p = P(non-dormant | A_1). We obtain P(non-dormant | A_1) by drawing it from a beta distribution, using α_{null} as the value for both α and β parameters. We then assigned the remaining n_{A1} - k copies of A_1 to the dormant group.

(iii) To fill out the remainder of the 2 x 2 table, we repeated this procedure for the second allele, A_2, drawing a new value from the same beta distribution to represent P(non-dormant | A_2).

Then, we performed the adjusted chi-squared test described in the main text on the resulting 2x2 tables, yielding a p-value. The null distribution evaluated for each α parameter consisted of p-values generated for all ~3.85 million SNPs in this way.

To select an appropriate α value to use for the FDR correction, we generated these null-distributions under a range of different values for α. We evaluated the fit of these null distributions to the real data by taking the chi-square distance between the null and observed p-value distributions. For this we used counts obtained from the *hist* function in R (R Core Team 2020), with 1000 identical bins used for all comparisons. Examining the fit of the whole range of p-values yielded a conservative FDR cutoff, as it is reflected in the Q-Q plot (Figure S3).

Finally, we estimated the FDR for the most significant SNPs. To do this, we generated the equivalent of 10 data sets under the null using the best-fit a value. Simulation of multiple data sets is standard when the FDR is obtained by simulation (Hastie et al. 2009), as the FDR is estimated using the values in the extreme tails of the null distribution. Since these samples are rare, simulating multiple data sets helps ensure that enough tail samples are obtained for an accurate FDR correction. The FDR for the ith ranked SNP was calculated by obtaining the number of simulated SNPs with lower p-values than this SNP and adjusting this number to reflect the average number obtained in sample of the same size as the real data set (i.e. dividing by 10 in this case). This represents the average number of SNPs from the null

distribution that are expected to have a value at least as extreme as that of the real SNP. This average is then divided by the rank of the SNP to obtain the FDR (e.g. for the p-value corresponding to the SNP with rank 10 from the data, if an average of 0.5 simulated *p*-values are equal to it or smaller, the FDR for that SNP is 0.5/10 = 0.05).

Library preparation and sequencing of individual flies

Genomic DNA of individual flies was extracted as described above. The NEBNext Ultra II FS DNA Library Prep kit was downscaled to 10% of the original reaction volume to prepare paired-end libraries from 70 ng genomic DNA. Size selection targeted an insert size of 350 bp. Libraries were amplified with 8 PCR cycles and sequenced using a 2 x 125 bp protocol on one lane of a HiSeq 2500 (Table S6). For trimming, mapping and downstream filtering we followed the pipeline described above.

References

Bastide, H., A. Betancourt, V. Nolte, R. Tobler, P. Stöbe, A. Futschik, and C. Schlötterer. 2013. A Genome-Wide, Fine-Scale Map of Natural Pigmentation Variation in Drosophila melanogaster. PLoS Genet. 9:e1003534.

Gómez-Sánchez, D., and C. Schlötterer. 2018. ReadTools: A universal toolkit for handling sequence data from different sequencing platforms. Mol. Ecol. Resour. 18:676–680.

Hastie, T., R. Tibshirani, and J. Friedman. 2009. Section 16.7 Feature Assessment and the Multiple-Testing Problem. P. *in* The Elements of Statistical Learning.

Kofler, R., R. V. Pandey, and C. Schlötterer. 2011. PoPoolation2: Identifying differentiation between populations using sequencing of pooled DNA samples (Pool-Seq). Bioinformatics 27:3435–3436.

Li, H., B. Handsaker, A. Wysoker, T. Fennell, J. Ruan, N. Homer, G. Marth, G. Abecasis, and R. Durbin. 2009. The Sequence Alignment/Map format and SAMtools. Bioinformatics 25:2078–2079.

Miller, S. A., D. D. Dykes, and H. F. Polesky. 1988. A simple salting out procedure for extracting DNA from human nucleated cells. Nucleic Acids Res. 16:1215.

Palmieri, N., V. Nolte, J. Chen, and C. Schlötterer. 2015. Genome assembly and annotation

of a Drosophila simulans strain from Madagascar. Mol. Ecol. Resour. 15:372–381.

Quesneville, H., C. M. Bergman, O. Andrieu, D. Autard, D. Nouaud, M. Ashburner, and D. Anxolabehere. 2005. Combined evidence annotation of transposable elements in genome sequences. PLoS Comput. Biol. 1:0166–0175.

R Core Team. 2020. R: A Language and Environment for Statistical Computing.

Smit, A., R. Hubley, and P. Green. n.d. RepeatMasker Open-4.0. http://www.repeatmasker.org 2013–2015.
