# Supplementary material for: Pool-GWAS on reproductive dormancy in Drosophila simulans suggests a polygenic architecture
Source: G3 (Bethesda). 2022 Feb 7;12(3):jkac027. doi: 10.1093/g3journal/jkac027 (PMC8895979; doi:10.1093/g3journal/jkac027)
Supplement: jkac027_Supplementary_File_3 [file jkac027_supplementary_file_3.pdf]

Pool-GWAS including regions with high coverage

We searched for structural polymorphisms associated with the trait. We reasoned that regions appearing in multiple copies in our sample data may collapse on each other on the reference assembly if only one copy is present in the assembly. This would lead to differences in coverage and allele frequencies. To search for such differences, we repeated the association analysis pipeline described in the main text, but omitted the RepeatMasker step and included positions with high coverage. We identified differences in coverage by searching for genome-wide differences in the mean coverage of 200bp windows between the two extreme dormancy groups (R package poolSeq, functions read.sync and coverage). Differences in allele frequencies were detected by the adjusted chi-squared test described in the main text. This alternative analysis unraveled two new regions with large differences in coverage between the two dormancy groups (up to 101) and very low p-values ($< 10^{-13}$): one region in chromosome X and one region in chromosome 2R (Figure S10 & Table S5).

The first region (chromosome X) corresponds to the 5'UTR of the long isoform of the gene *Trf2* (*TATA box binding protein-related factor 2*) (Figure S13). *Trf2* expression is enriched in reproductive organs, participates in cold acclimation (MacMillan et al. 2016) and ecdysone-induced developmental programmed cell death (Bashirullah et al. 2007; Wang et al. 2008). In *Trf2*, the Non-Dormant group has higher coverage in this region compared to the Dormant group. We reconstructed the haplotype that creates this excess of coverage and searched its identity through blast. Interestingly, it corresponds to the *rIST* (rearranged *IST*), a haplotype emerging from tandem duplication of a large region in *D. simulans* that leads to the Paris *Sex-Ratio* Drive (Fouvry et al. 2011; Bastide et al. 2013). The duplicated region contains six genes, from *Trf2* to *Org-1*, the *rIST* is similar in sequence to the duplication breakpoint region of *Trf2* and appears in alternating tandem repeats together with the *HOSIM1* transposable element between the two copies of the duplicated region (Figure S13). Since the reference assembly lacks this large duplication, the *rIST* copies map on the breakpoint region of *Trf2,* creating the excess of coverage we observed. The second region is located in the beginning of 2R (Figure S10) and is directly linked to *Trf2* and the excess of coverage associated with it. This region in 2R does not contain any gene and is badly assembled, but the high-association SNPs are found together on a single segment (Figure S14). We blasted this segment and it corresponds to the *HOSIM1* transposable element, which is present in the Paris *Sex-Ratio* Drive genotype.

We reasoned that, since we used Pool-Seq data, copy number variation in a few individuals from a pool can already result in a moderate coverage increase. To investigate whether the observed copy number differences between the two groups were the result of such an artifact, we sequenced single phenotyped female flies from each of 12 strains from the Non-Dormant group. We chose flies with the non-dormant phenotype for individual sequencing since the regions with high coverage described above were specific to the Non-Dormant group. Sequencing single flies identified coverage heterogeneity among individuals in these regions. In fact, only a single fly (strain SS1294) had high coverage at the breakpoint region of *Trf2* (Figure S11) and the *HOSIM1* segment in the beginning of chromosome 2R (Figure S12). For this reason, we did not further pursue structural variation as a major contributor to dormancy variation.

References

Bashirullah, A., G. Lam, V. P. Yin, and C. S. Thummel. 2007. *dTrf2* is required for transcriptional and developmental responses to ecdysone during *Drosophila* metamorphosis. Dev. Dyn. 236:3173–3179.

Bastide, H., P. R. Gérard, D. Ogereau, M. Cazemajor, and C. Montchamp-Moreau. 2013. Local dynamics of a fast-evolving sex-ratio system in *Drosophila simulans*. Mol. Ecol. 22:5352–5367.

Fouvry, L., D. Ogereau, A. Berger, F. Gavory, and C. Montchamp-Moreau. 2011. Sequence analysis of the segmental duplication responsible for paris sex-ratio drive in *Drosophila simulans*. G3 Genes, Genomes, Genet. 1:401–410.

MacMillan, H. A., J. M. Knee, A. B. Dennis, H. Udaka, K. E. Marshall, T. J. S. Merritt, and B. J. Sinclair. 2016. Cold acclimation wholly reorganizes the *Drosophila melanogaster* transcriptome and metabolome. Sci. Rep. 6:28999.

Wang, L., J. Evans, H. K. Andrews, R. B. Beckstead, C. S. Thummel, and A. Bashirullah. 2008. A genetic screen identifies new regulators of steroid-triggered programmed cell death in drosophila. Genetics 180:269–281.
