# Supplementary material for: Pool-GWAS on reproductive dormancy in Drosophila simulans suggests a polygenic architecture
Source: G3 (Bethesda). 2022 Feb 7;12(3):jkac027. doi: 10.1093/g3journal/jkac027 (PMC8895979; doi:10.1093/g3journal/jkac027)
Supplement: jkac027_Supplementary_Data [file jkac027_supplementary_data.zip › jkac027_Supplementary_Figure_S13.pdf]

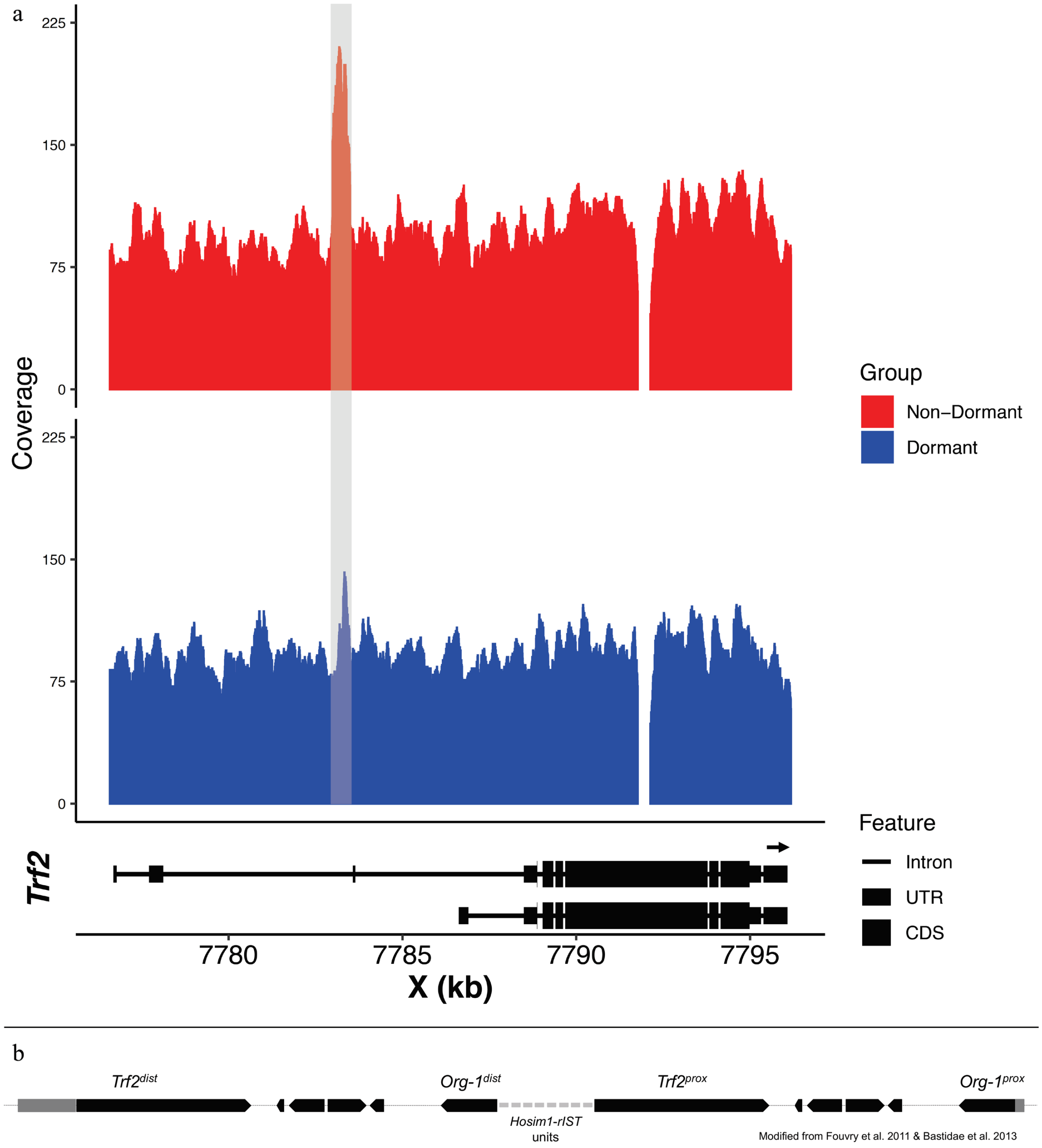

Figure S13: a) Coverage of the *Trf2* region on chromosome X that exhibits high coverage (shadowed area). b) A region spanning six genes on the X chromosome is duplicated and creates the Paris *Sex-Ratio* Drive. Between the two copies lie alternating tandem repeats of the *HOSIM1* transposable element and the *rIST* sequence. The latter is similar in sequence to the duplication breakpoint region of *Trf2* and the copies of it create the excess of coverage in the *Trf2* region.
