# Supplementary material for: Pool-GWAS on reproductive dormancy in Drosophila simulans suggests a polygenic architecture
Source: G3 (Bethesda). 2022 Feb 7;12(3):jkac027. doi: 10.1093/g3journal/jkac027 (PMC8895979; doi:10.1093/g3journal/jkac027)
Supplement: jkac027_Supplementary_Data [file jkac027_supplementary_data.zip › jkac027_Supplementary_Figure_S14.pdf]

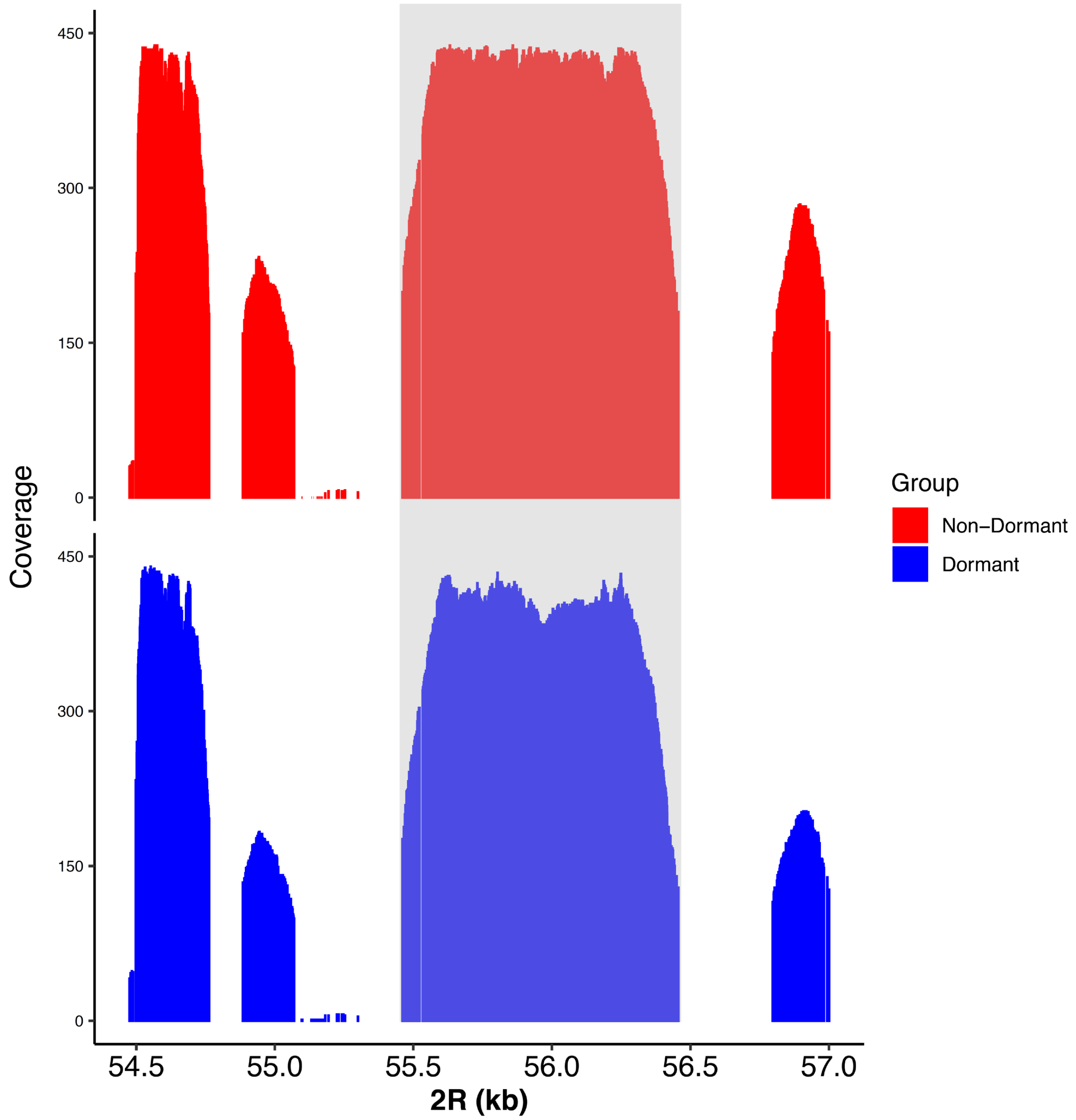

Figure S14: Coverage of the region in the beginning of 2R that gave a strong association signal. This region does not contain any gene and is badly assembled. The high-association SNPs are found together on a single segment (shadowed area). This segment corresponds to the *HOSIM1* transposable element, which is present in the Paris *Sex-Ratio* Drive genotype.
